# Malaria case management and elimination readiness in health facilities of five districts of Madagascar in 2018

**DOI:** 10.1186/s12936-020-03417-z

**Published:** 2020-10-01

**Authors:** Anjoli Anand, Rachel Favero, Catherine Dentinger, Andrianandraina Ralaivaomisa, Sitraka Ramamonjisoa, Oliva Rabozakandraina, Eliane Razafimandimby, Jocelyn Razafindrakoto, Katherine Wolf, Laura Steinhardt, Patricia Gomez, Malanto Rabary, Mauricette Nambinisoa Andriamananjara, Sedera Aurélien Mioramalala, Jean-Pierre Rakotovao

**Affiliations:** 1 Epidemic Intelligence Service, Centers for Disease Control and Prevention, 1600 Clifton Road, Atlanta, GA 30333 USA; 2 Malaria Branch, Division of Parasitic Diseases and Malaria, Center for Global Health, Centers for Disease Control and Prevention, Atlanta, GA USA; 3 Maternal Child Survival Program, Washington, DC USA; 4 US President’s Malaria Initiative, US Centers for Disease Control and Prevention, Antananarivo, Madagascar; 5 Maternal Child Survival Program, Antananarivo, Madagascar; 6 Independent Consultant, Antananarivo, Madagascar; 7 National Malaria Control Programme, Ministry of Health, Antananarivo, Madagascar

**Keywords:** Malaria elimination, Malaria control programme, Health facility survey

## Abstract

**Background:**

Madagascar’s Malaria National Strategic Plan 2018–2022 calls for progressive malaria elimination beginning in low-incidence districts (< 1 case/1000 population). Optimizing access to prompt diagnosis and quality treatment and improving outbreak detection and response will be critical to success. A malaria elimination readiness assessment (MERA) was performed in health facilities (HFs) of selected districts targeted for malaria elimination.

**Methods:**

A mixed methods survey was performed in September 2018 in five districts of Madagascar. Randomly selected HFs were assessed for availability of malaria commodities and frequency of training and supervision conducted. Health providers (HPs) and community health volunteers (CHVs) were interviewed, and outpatient consultations at HFs were observed. To evaluate elimination readiness, a composite score ranging from 0 to 100 was designed from all study tools and addressed four domains: (1) resource availability, (2) case management (CM), (3) data management and use, and (4) training, supervision, and technical assistance; scores were calculated for each HF catchment area and district based on survey responses. Stakeholder interviews on malaria elimination planning were conducted at national, regional and district levels.

**Results:**

A quarter of the 35 HFs surveyed had no rapid diagnostic tests (RDTs). Of 129 patients with reported or recorded fever among 300 consultations observed, HPs tested 56 (43%) for malaria. Three-quarters of the 35 HF managers reviewed data for trends. Only 68% of 41 HPs reported receiving malaria-specific training. Of 34 CHVs surveyed, 24% reported that treating fever was no longer among their responsibilities. Among treating CHVs, 13 (50%) reported having RDTs, and 11 (42%) had anti-malarials available. The average district elimination readiness score was 52 out of 100, ranging from 48 to 57 across districts. Stakeholders identified several challenges to commodity management, malaria CM, and epidemic response related to lack of training and funding disruptions.

**Conclusion:**

This evaluation highlighted gaps in malaria CM and elimination readiness in Madagascar to address during elimination planning. Strategies are needed that include training, commodity provision, supervision, and support for CHVs. The MERA can be repeated to assess progress in filling identified gaps and is a feasible tool that could be used to assess elimination targets in other countries.

## Background

Renewed interest in malaria elimination in the 21st Century is due in part to advances in diagnostic tools, anti-malarial therapy, and vector control strategies that have led to reductions in malaria burden over the last 20 years [1]. Previous efforts at malaria eradication by the Global Malaria Eradication Programme were not successful in sub-Saharan Africa for a variety of reasons [2, 3].

In 2007, Bill and Melinda Gates called for a malaria eradication agenda [4]. This aspiration was quickly adopted by World Health Organization (WHO) and inspired health officials in many malaria-endemic countries, including those in sub-Saharan Africa, to prioritize the transformation of malaria control programmes into elimination programmes. The WHO does not identify a specific threshold at which to begin elimination activities, recommending instead that different goals be applied sub-nationally and in accordance with the transmission setting [5]. Various working groups have identified programmatic activity priorities for elimination contexts, such as surveillance-response approaches that align with recommended WHO activities [6, 7]. While malaria elimination activities are context specific, they fall under broad categories of strategies that include: enhancing and optimizing case detection and management, local stratification by malaria transmission intensity, and surveillance incorporating routine data in a timely and reliable manner [5].

Madagascar is one of many countries with endemic malaria but variable transmission zones, including some where government officials and malaria stakeholders are developing plans for sub-national elimination [8]. In 2016, malaria was responsible for approximately 5.9% of outpatient visits, 4913 health centre admissions, and 6.7% of deaths; however, in some districts, primarily those of the Central Highlands, a high-altitude area with historically low transmission, malaria incidence was < 1 case/1000 population/year, which meets the National Malaria Control Programme (NMCP) criteria for elimination planning [X, 8]. Criteria for pre-elimination planning includes an incidence of 1-10 cases/1000 population/year plus a health facility test positivity rate < 5%. [8].

During 2018, the NMCP began work on its malaria elimination strategy based on WHO guidance and in collaboration with Roll Back Malaria partners and stakeholders [5, 16]. To inform this strategy, the NMCP sought to determine the malaria elimination readiness of the health system and its ability to perform and sustain elimination activities. Currently, there are broad guidelines for types of activities an elimination programme should contain depending on local context and needs. However, there are few tools or guidelines for determining where or how to direct efforts and resources in planning elimination targets and activities at the beginning of the process. Equally scare are guidelines on how to evaluate a country’s readiness to undertake elimination initiatives, e.g., in specific geographic areas. Given the lack of standardized assessment tools to determine malaria elimination readiness, the study team used available materials to design a suite of tools based on recent experiences in countries, such as Ethiopia, as well as previous efforts to quantify health system readiness and service delivery more generally [5, 9–14, 16]. The objectives of the assessment were to conceptualize an approach to evaluating elimination readiness in health facilities appropriate for the Madagascar context, conduct a comprehensive survey in selected elimination districts of Madagascar, and use this approach to analyse and present findings on elimination readiness in these districts to inform elimination planning, including surveillance for elimination, in Madagascar.

## Methods

### Study setting and design

**Fig. 1:**
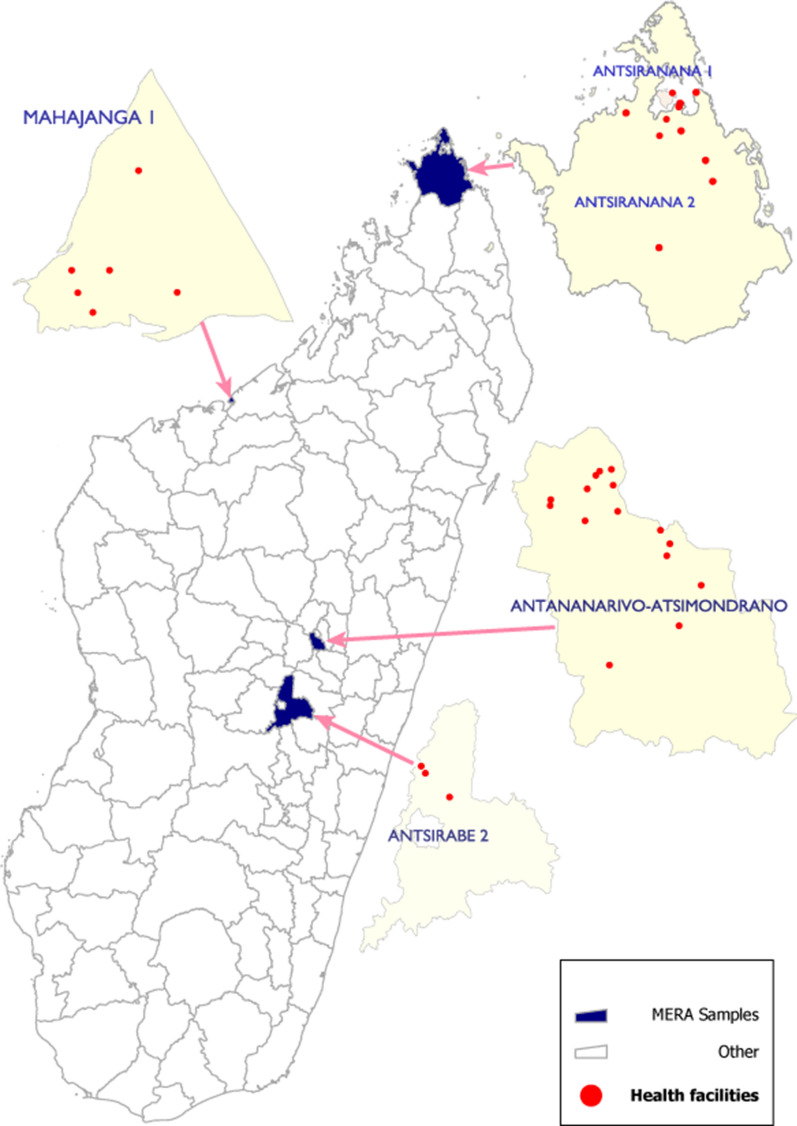
Map of Madagascar showing the location of health facilities selected for the malaria elimination readiness assessment by district (2018)

The malaria elimination readiness assessment (MERA) was designed as a component of a nationwide health facility assessment (HFA), a mixed-methods cross-sectional survey designed to evaluate febrile illness and malaria CM quality [15]. The MERA component included an expanded protocol to assess the readiness of Madagascar’s health system to undertake a malaria elimination programme; the target population for MERA included the district residents, clinicians and public health system. Four of eight districts in Madagascar meeting the NMCP criteria for elimination or pre-elimination using 2016 routine malaria surveillance data [X, 8] were purposively selected to include the MERA; these districts were Antananarivo-Atsimondrano, Mahajanga I, Antsiranana I, and Antsirabe II (Fig. 1) [8]. In addition, a fifth district, Antsiranana II, which qualified as an elimination district based on 2017 routine data, was added during data collection because survey teams were unable to achieve the targeted sample of outpatient consultations in Antsiranana I. The MERA protocol included all elements of the HFA: quantitative data were collected via a health facility (HF) assessment, a survey of healthcare providers (HPs), and clinical observations of HPs and patients; qualitative data were collected via discussions with stakeholders at the national, regional, and district levels, and structured surveys of community health volunteers (CHVs) who were supervised by the sampled HFs.

### Sampling approach

Sampling for the MERA followed that of the HFA, which was designed to provide a national estimate of the proportion of febrile patients who received a malaria diagnostic test [15]. For sampling of the elimination districts, in the four HFA survey zones (North East, North West, Central Highlands, and Highland Fringe East) with at least one elimination district, an elimination district was selected (randomly if more than one). The number of HFs sampled per zone was proportional to the number of public and private HFs in that zone. One private HF was randomly selected per district with the remainder selected among public facilities for a total of four private and 31 public HFs. One to two HPs present on the day of the scheduled survey at each facility were invited to participate; the senior provider was approached first, in the event a facility had additional providers, a second randomly-selected provider was invited to participate. In addition, one randomly selected CHV who was associated with that facility (Table 1). Qualitative data were collected through key informant interviews with respondents from the national level (NMCP, commodities management agency, case management support partners), regional level (malaria directors in the four regions), and district levels (district malaria officers in the elimination districts) (Table 2).

**Table 1:** Districts, health facilities, clinicians, patient consultation observations, and community health volunteers included in the final sample of the MERA

Malaria Operational Zone	District	HF (Public)	HF (Private)	Tool 1: HF Checklist	Tool 2: HP Interview	Tool 3: HP-Patient Observation	Tool 5: CHV Interview*
Central Highlands	Antsirabe II	2	1	3	5	20	3
Highland Fringe East	Antananarivo-Atsimondrano	14	1	15	18	134	14
Northwest	Mahajanga I	5	1	6	6	54	5
Northeast	Antsiranana I	3	1	4	4	36	3
Northeast	Antsiranana II	7	0	7	8	56	9
Total	5	31	4	35	41	300	34

*CHVs were not available to participate at all health facilities

**Table 2:** Stakeholders interviewed about perspectives on malaria case management and elimination programme needs

	Stakeholder interviewed	#
Ministry of Health	NMCP Official	1
	Regional malaria officer	5
	Malaria district officer, medical Inspectors, or technical assistant	7
Technical/financial partners	Coordinator of vertical programme, malaria commodity purchasing	1
	Malaria case management officer, non-governmental organization	1
	Malaria officer, non-governmental organization	1
	Distribution manager, procurement and supply management project	1
	Total	17

### Data collection

Quantitative survey tools were developed based on elimination planning tools designed in Ethiopia and Laos [unpublished materials], the WHO Service Availability and Readiness Assessment (SARA) checklist, and WHO malaria elimination guidance documents [5, 10, 16]. Four structured, quantitative questionnaires, including an HF checklist, surveys for HPs and CHVs, and observation of an HP outpatient visit gathered information on elements that would be required to perform activities relevant to achieving malaria elimination. These included availability of commodities, recent stock-outs, frequency of supervision visits, availability of technical assistance from higher levels within the health system, data reporting and usage procedures, and outbreak response experience. These tool elements then fed into the overall malaria elimination scoring system (Table 3; additional details in Additional file 1: Table S1). A data collection training workshop was held to review the surveys, resolve differences in interpretation, and standardize the observation scoring among data collectors. Data collectors field-tested the tools in a facility that was not selected for the survey, and adjustments were made to the tools based on their feedback. Survey teams consisted of three trained team members. On the day of the survey team’s visit, the HF checklist was completed by the highest-ranking person at the facility. Structured questionnaires were administered to a sampled HP providing clinical care and one randomly sampled CHV associated with the facility about their experiences and perspectives on challenges to providing high quality case management, data use, training, and supervision. Interactions between HPs and patients visiting the facility were observed. In HFs with fewer than 20 patients expected on that day, every patient was invited to participate, otherwise every other patient was approached. The number of patients expected to visit the facility during the study team’s visit was determined by reviewing the register entries for the same day in the previous week. During the consenting process, patients were asked by the study team if they had had a febrile illness in the previous 48 h and the response was documented. During the clinical consultation, surveyors recorded patients’ spontaneous reporting of fever, HPs’ ascertainment of fever, testing for malaria, diagnosis given, and prescribing anti-malarial medications as appropriate per guidelines. In the event a surveyor observed incorrect care, they discretely informed the HP after the observed clinical encounter so that no RDT-positive patient left the HF without proper treatment. The survey teams brought a stock of artemisinin-based combination therapy (ACT) in case of HF stock-out. CHVs were surveyed about their experiences and perspectives on case management, data use, training, and supervision.

**Table 3:** Select indicators and survey questions included in the malaria elimination readiness score by domain for MERA survey

Categories of indicators	Questions included
*Domain 1: Resource availability*	
Diagnostic capacity	Malaria RDTs in stock^a^ Able to diagnose *P. vivax*^b^
Essential medicines	Either ACT or oral quinine in stock^a^ Either injectable artesunate or quinine in stock^a^ Primaquine in stock^a^
Other commodities	Thermometer available^a^ Infant weighing scale available^a^ Stand-on scale available^a^
Guidelines	Copy of National Malaria Guidelines available^a^
CHV testing and diagnosis	Thermometer available^c^ RDT currently available^c^ ACT currently available^c^ No stock-outs in past 3 months^c^
Stock management system	Stock management system being used^a,c^
*Domain 2: Case management*	
CHV patient population	CHV treats children ≤ 5 years with fever^c^
Identifying criteria for testing for malaria	HP identifies criteria for testing for malaria^b^ CHV identifies criteria for testing for malaria^c^ HP elicits fever complaint^d^ HP takes patient’s temperature^d^ HP performs RDT in patient with fever complaint^d^
Test and treating practices	RDTs performed for reported fever in absence of current fever^c,d^ Patients not treated without testing^b^ ACT given for positive RDT^c^
Management of uncomplicated malaria	HPs use appropriate anti-malarials^b^ CHVs follow-up with patients after treatment^c^
Accessing high risk populations	CHVs assess travel history and encourage pregnant women to seek care^c^
SBC activities	Malaria education is performed in the community^a,c^
*Domain 3: Data management and use*	
Data reporting	Case data records are maintained^c^ Data are reported to HFs^c^
Data analysis	Standard operating procedures (SOP) available for data analysis^a^ Data reviewed monthly^a^ Data used to make decisions^a^ Case location can be mapped geographically^a^
Data quality	Cases classified as confirmed or clinical^a^ Cases can be followed across registers^a^ Data quality measures in place^a,c^ Data quality is monitored^a^ Monthly summary reports are maintained^c^
Epidemic response	Actions are undertaken in the event of increase in cases above expected^a,c^
*Domain 4: Training, supervision and technical assistance*	
Training	HFs and CHVs report receiving training on various malaria activities and data management^a,b,c^
Supervision	HFs and CHVs report receiving supervision visit in the last 6 months^a,c^
Feedback	HFs and CHVs report receiving feedback on data^a,c^
Technical assistance	HFs and CHVs report receiving technical assistance from higher level if requested^a,c^ HFs and CHVs report receiving guidance on malaria outbreak response^a,c^

^a^Health Facility (HF) assessment

^b^Healthcare Provider (HP) interview

^c^Community Health Volunteer (CHV) interview

^d^Patient observation

A structured interview guide was developed for the qualitative survey and administered to key stakeholders identified by the research team (Table 2). Stakeholders were asked questions about challenges they perceived in the implementation of malaria case management, maintaining high quality surveillance, supply chain management, epidemic response, and transforming the malaria control programme into an elimination-oriented programme.

The survey was conducted in September 2018. Responses were recorded on paper forms and entered into CSPro 7.1 (US Census Bureau, Washington DC, USA). During data collection, supervisors reviewed data daily to ensure quality and completeness. Qualitative data were recorded in audio format, transcribed, and translated from Malagasy into French for analysis. Results are presented in English. All respondents for all tools were given codes; links to personal identifiers were destroyed. The data were stored in password-protected cloud-based Microsoft Word documents. Survey tools are available in Additional file 2: Survey Tool.

### Analysis

The study team used descriptive statistics to analyse study data from all four quantitative assessment tools. In addition, a health system malaria elimination readiness score was developed based on the approaches of the WHO SARA tool which includes both facility-and individual-provider level data [10]. Preparing the domain components and the corresponding scoring system for this assessment was an iterative process that occurred over the course of several months. Components of the quantitative surveys were grouped under four domains relevant to malaria elimination programming: (1) resource availability; (2) CM; (3) data management and use; and, (4) training, supervision and technical assistance. Each question relevant to a certain domain was assigned a value of one point. Responses within questions with “select all that apply options” were given fractional points that summed to one. The scoring guide developed is available in Additional file 2. The HF was the unit of analysis. In situations where more than one HP or CHV per facility was interviewed, their scores were averaged. Data from clinical observations were included under the CM domain. As survey teams completed varying numbers of clinical observations, observation-specific sub-scores were calculated and then averaged for each facility. Given the importance of fever identification and testing practices for malaria elimination, the score for the CM domain was weighted so that clinical observation data accounted for half its total. Each domain score, as well as a final score that was a simple average of the domains, was scaled to an absolute number from 0 to 100. Additional details on the scoring guide are presented in Additional file 3: Scoring Tool.

The aim of the qualitative data analysis was to illuminate key themes found throughout the transcripts related to elimination readiness and planning. A thematic analysis approach was used, linking identified themes to codes developed before or during analysis as new themes emerged [17]. Data were cleaned and analysed using Microsoft Excel (Microsoft Corp, Washington, USA), SAS 9.4 (SAS Institute Inc., North Carolina, USA), and STATA 14 (StataCorp LLC, Texas, USA).

## Results

Quantitative data were collected from 31 public HFs and four private HFs, 41 facility-based HPs (37 public and four private), 34 CHVs, and 300 clinical observations of HPs conducting outpatient consultations at HFs across the five districts (Table 1). Surveyed HPs included doctors (76%), nurses (12%) and midwives (12%). All HPs and CHVs had been practicing at or associated with their HF for at least 6 months. HFs included 32 (91%) basic health centres, one (3%) hospital, one (3%) medical office and one (3%) free medical centre; only outpatient services were assessed. The range of clinical outpatient observations per HF was 1–12, with an average of nine. The wide range in observations was due to low numbers of patient visits on the day of the study team’s visit. Children under 5 years of age accounted for 31% of observations. Qualitative data were collected during 17 stakeholder interviews (Table 2).

### Domain scores for four domains

The average elimination readiness score across the four domains in all five districts was 52 out of 100, ranging from 48 to 57 per district (Table 4). Although district scores were similar, facility scores ranged widely from 30 to 61. For example, Antananarivo-Atsimondrano district includes facilities scoring between 30 and 61 (Table 4; additional details in Additional file 4: Table S2). Domain-specific scores were lowest for training, supervision and technical assistance, with an average of 40, and highest for data management and use, with an average of 63. There was also a wide range of scores between facilities within a district for specific domains.

**Table 4:** District-level malaria elimination readiness scores and domain-level scores (out of 100), mean (range)

District	Resource availability	Case management	Data management and use	Training, supervision and assistance	Total
Antsiranana II	67 (64–72)	43 (32–56)	68 (63–77)	50 (34–58)	57 (54–61)
Antsiranana I	64 (44–78)	55 (52–60)	57 (40–66)	40 (21–62)	54 (47–61)
Mahajanga I	50 (39–69)	51 (44–62)	62 (53–77)	47 (18–67)	53 (50–58)
Antsirabe II	56 (46–61)	48 (39–54)	65 (61–72)	34 (23–51)	51 (45–59)
Antananarivo–Atsimondrano	57 (44–78)	42 (27–57)	62 (21–90)	32 (11–52)	48 (30–61)
Total sample population	58 (39–78)	46 (27–62)	63 (21–90)	40 (11–67)	52 (30–61)

### Domain 1: Resource availability

This domain catalogued the availability of various commodities necessary to evaluate fever and diagnose and treat malaria based on national guidelines. Nearly a quarter of HFs had no rapid diagnostic tests (RDT) at the time of the survey (Table 5). An age-appropriate course of the first-line anti-malarial therapy, artesunate-amodiaquine (ASAQ), was available in at least 80% of HFs, and 43% had primaquine in stock for single low-dose gametocytocidal therapy. A third of HFs had either injectable artesunate or injectable quinine in stock for severe malaria (Table 5). About a third of HPs (34%) reported that providers in their HF are able to test for *Plasmodium vivax,* and 24% of HPs report doing so. However, all HFs only had multi-species RDTs, no functional microscopes. Over half (56%) of CHVs surveyed had no malaria commodities; upon further questioning, eight of the 34 CHVs reported that they no longer provide any CM services though they continue to provide education and refer febrile patients to HFs.

**Table 5:** Performance of all HFs, HPs, and CHVs in select indicators by MERA domain

	HFs	HPs	Observations	CHVs
*Domain 1: Resource availability, n/N (%)*				
Thermometers in stock	31/35 (89)			3/34 (9)
RDTs in stock	27/35 (77)			13/26 (50)
ASAQ in stock	28/35 (80)			11/26 (42)
Primaquine in stock	15/35 (43)			
Injectable artesunate or quinine in stock	12/35 (34)			
RDT stock out in past 2 months	9/35 (26)			
Providers are able to test for *P. vivax*		14/41 (34)		
Providers diagnose *P. vivax* in their facility		10/41 (24)		
ACT (age 14 +) stock out in past 2 months	10/35 (29)			
ACT (age 5–13) stock out in past 2 months	8/35 (23)			
Stock out of any RDTs and ACTs at time of survey				19/34 (56)
*Domain 2: Case management, n/N (%)*				
Identifies history of fever as criteria for suspect case		34/41 (83)		18/26 (69)
Identifies history of any symptoms in recent traveler as criteria for suspect case		29/41 (71)		5/26 (19)
Asked fever history if patient did not spontaneously report			92/189 (49)	
Took temperature of patient			195/300 (65)	
A temperature was recorded			142/300 (47)	
Performed RDT for patient with reported or recorded fever			56/129 (43)	
Patient with positive RDT was given an ACT			4/5 (80)	
Reports asking for travel history				27/34 (79)
Reports performing RDT if patient has history of fever				20/26 (77)
Reports giving ACT for positive RDT				12/26 (46)
*Domain 3: Data management and use, n/N (%)*				
Uses a registry to record consultations				30/34 (88)
Reviews monthly data for trends	26/35 (74)			
Able to follow individual patients between registers (ex: laboratory and pharmacy registers)	19/35 (54)			
Able to map cases geographically	24/35 (69)			
Use data to make decisions (ex: community outreach, request assistance or commodities)	25/35 (71)			18/34 (53)
Ever received guidance on how to interpret and use data	9/35 (26)			
Reviews registers for data quality	33/35 (94)			
Ever received guidance on how to assess data quality	8/35 (23)			
Performs active case detection or community level outreach in the event of outbreak	2/35 (6)			
Able to perform community outreach in the event of an outbreak				30/34 (88)
*Domain 4: Training, supervision, technical assistance, n/N (%)*				
Received malaria specific training		28/41 (68)		
Received malaria elimination training		6/41 (15)		
Received supervision vision in past 6 months	15/35 (43)			20/34 (59)
Received data management training in past 2 years	10/35 (29)			19/34 (56)
Data quality audit was performed in past year	12/35 (34)			
Reported ever receiving feedback on submitted data	25/35 (71)			9/34 (26)
Received guidance on how to respond to an outbreak	11/35 (31)			17/34 (50)

### Domain 2: Malaria case management

The CM domain assessed HPs’ and CHVs’ ability to identify a suspected case of malaria and execute the appropriate clinical and diagnostic examination once identified. Although the definition of a suspected case is not explicitly stated in the NMCP’s guidelines, the Madagascar malaria treatment protocols, per officials interviewed for this survey, include testing all fevers with a malaria diagnostic test and treatment of uncomplicated malaria with a combination of ACT and single low-dose primaquine. Among HPs surveyed, 83% mentioned that history of fever is a criterion for testing for malaria (Table 5). During HP-patient observations, the HP asked for a fever history in 49% of interactions with patients who did not spontaneously report a fever. The patient’s temperature was measured in 65% of all interactions. Fewer than half (43%) of patients who met the criteria for testing based on actual or reported fever were tested with an RDT. Among the 27 HFs that reported having RDTs in stock on the day of the survey, 48% of patients who met criteria for testing received an RDT. The RDT was positive in five consultations, and ACT was given to four of those patients; amoxicillin was prescribed to the fifth patient. The study team ensured the patient was given ACT prior to departure per study protocol. One patient with a negative RDT was given ACT. Among CHVs surveyed, 77% reported that they perform an RDT if a patient has a history of fever and 46% of CHVs reported that they would provide ACT for patients with positive RDTs.

### Domain 3: Data management and use

This domain captured the data reporting procedures and use of HF managers and CHVs. Three-quarters of HF managers reviewed data monthly on their own for trends, and 71% reported using malaria data to make decisions including resource allocation, improving surveillance indicators and identifying training needs (Table 5). Nearly all managers reported reviewing the quality of the data in their registers. Use of a patient register was reported by 88% of CHVs, and 53% reported using it to make decisions. However, funding constraints stymied timely submission of data. One CHV reported, *“I have not sent another report since July because we have not received our allowance* [for transport to HFs] *since then.”* About 69% of managers reported that they could map cases geographically. When asked how the HF responds to an unexpected increase in malaria cases, 6% of managers reported performing an investigation or community level outreach to test and treat; none recruited CHVs to assist with the response. The majority (88%) of CHVs surveyed reported the ability to perform educational outreach activities if an unexpected increase in cases occurs.

### Domain 4: Training, supervision and technical assistance

HPs and CHVs were asked about the frequency and types of training, supervisory and technical assistance visits they had received. Technical assistance refers to situations wherein higher levels of the health system address issues experienced by the HF, such as increases in malaria cases or commodity stock-out. Sixty-eight per cent of HPs reported receiving malaria-specific training in the previous 2 years. A supervisory visit for malaria CM was performed within the previous 6 months in 43% of HFs (Table 5). Among CHVs, 59% had received a supervisory visit within the past 6 months. A data quality audit was performed within the previous year in 34% of HFs, and 71% of HF managers reported having received any kind of feedback from the district on submitted data. About a quarter of CHVs reported receiving feedback on malaria CM indicators in the previous 3 months. A third of facility managers and 50% of CHVs reported receiving guidance on how to respond to a malaria outbreak.

### Stakeholder interview

#### Commodity availability and use

Stakeholders identified several potential reasons for the gaps in commodity coverage noted above, including inadequate storage at the facility level. Due to staffing shortages, forecasting commodity needs is often performed by staff who fill other roles.

*“Human resources are a challenge. Most [HF] managers do not have the capacity to handle logistics. You need someone who has studied logistics to manage the commodities because it is not easy.”*—Technical and financial partner.

Most commodities are provided by donor organizations, and as such, termination of or other changes to donor agreements can disrupt services.

*“Funds come 97% [of the time] from donors. When [donor] funding is not available, activities cannot be implemented. For example, the [NMCP’s] 2013 IRS [indoor residual spraying] campaign could not be done. The [Madagascar] government’s share in all malaria control activities represents 7% of the total budget of [malaria control] services.”*—NMCP Official

One NMCP official noted that supply shortages may be particularly problematic for CHVs because they depend on support from non-government organizations (NGO) and HFs for supplies. When NGO funding shifts or ends, or HFs are unable to meet their own needs, there are frequently CHV-level stock-outs. When HFs cannot order and store adequate supplies for their own needs and those of multiple CHVs, they prioritize their own needs over those of CHVs.

Some interviewees raised concern that incorrect treatment was due to patient expectation, stating that some patients believe that a more costly treatment means better care. Furthermore, the potential for a health care worker to profit from an alternative medication was suggested as a driver of non-adherence to protocol rather than a lack of ACT medicines; because ACT is free, alternative medication that requires payment is sometimes preferred or given.

*“It’s better to prescribe a drug that costs more to [increase your profit].”*—District-level manager

Interviewed stakeholders also believe that some private-sector HPs do not believe in the effectiveness of subsidized drugs.

*“They think that a cheap drug is less effective than those that cost more, and they prefer to inject quinine, even for simple malaria.”*—Regional malaria officer

#### Supervision and training

Stakeholders acknowledged that HPs are overworked. Facilities are understaffed given the number of programmes they manage, each with its own reporting structure and funding source. Malaria testing and treatment errors may result from competing interests on providers’ time. This is exacerbated by inadequate training. Stakeholders noted that new staff do not always receive adequate training on malaria CM and reporting. Private sector HPs were thought to avoid trainings because leaving their post would result in a loss of income.

Stakeholders noted that regular supportive supervision could address training gaps. However, HFs can be very challenging for supervisors to reach and in some areas, safety [from bandits] is a concern.

*“One facility in my district is 150* *km away and can only be reached after a four*-*hour canoe ride followed by 2* *hours of walking. During rainy season, this area becomes completely cut*-*off because the river is [too] dangerous*—*not because of high water, but because of crocodiles.”*—District-level manage

District managers and technical/financial partners noted that supervisors do not have adequate funding to visit all facilities and must choose between them.

*“The [NMCP] provides limited funds for supervision so we only visit nearby facilities even though the need is greater in remote facilities.”* –District-level manager

Stakeholders identified a need for improved communication and coordination at all levels of the health system. In the absence of good communication, there can be redundancies in some places and gaps in others.

*“Central and regional staff conduct the same supervision in one place at the same time. This should be avoided because it is a waste of resources. Why is it that there is no programme integration, since a single facility is being supervised?”*—Regional manager

### Challenges to providing high quality case management

Stakeholders acknowledged that poor CM might be related to low HP wages and poor living and working conditions. Similarly, CHVs face barriers that can impact their motivation and ability to provide high quality care.

*“CHVs have responsibilities for social mobilization, census taking, and case management for children under 5, yet they are volunteers. They may lose motivation, as they are unpaid, and they sometimes do things wrong.”*—National-level programme manager

It was noted above that several CHVs surveyed reported being directed to stop providing clinical care for patients with malaria for reasons that are not immediately clear. They do, however, continue health promotion and malaria sensitization work in the community.

### Malaria elimination activities and plannin

When asked about malaria elimination activities and planning, there was a lack of consensus among stakeholders over the definition of an outbreak and how that threshold was set. The calculation of the epidemic threshold varied according to each respondent, and those not directly involved in programme implementation said they were unsure how to calculate it. Responses included “average + 2 standard deviations,” “average standard deviation of the past 5 years,” “average standard deviation of the past 3 years”, “doubling of cases during 2 successive weeks,” and “doubling of cases during 3 successive weeks.” Technical and financial partners who were not directly involved in programme planning did not have a sense of how the threshold was set. There was also a lack of consensus over which level of the health system was responsible for calculating the epidemic threshold: six respondents thought the HF determines the level; three respondents thought this was done at the sub-district commune level; six respondents thought it was the district; and two respondents thought it was the region. The frequency at which the epidemic thresholds are adjusted also varied according to respondents: on an annual basis, according to three respondents; monthly, according to one respondent; weekly, according to two respondents; and seasonally, according to one respondent, who added that the threshold should be lowered during the rainy season.

Respondents were able to identify the types of activities outlined in the National Strategic Plan for elimination zones. These included case confirmation with an RDT, treatment of confirmed cases with an ACT and low-dose primaquine, focused indoor residual spraying with insecticide in the event of an outbreak, focused coverage of at-risk populations with mosquito nets, weekly epidemiologic surveillance, strengthening of data collection and reporting, reactive case detection, community education, and efforts aimed at travellers and migrants.

*“If there is a doubling of cases, health workers conduct an investigation. If they cannot solve the problem, they notify the district, and depending on the severity of the situation, it will go up to the regional level, then to the central level.”*—Regional-level malaria manager

*“If the person is sick when he/she arrives [in the area], he/she is given treatment. But if he/she has been in the community more than a week before getting sick, we consider [that person] an index case and investigate malaria in the five houses surrounding the patient’s house.”*—District-level manager

These responses suggest that some level of reactive case detection is occurring though it is unclear what the trigger for an investigation is, given how variable the responses were to determining an epidemic threshold, or who is responsible for initiating the activity.

## Discussion

MERA was designed and applied to assess the current state of the health system in Madagascar with regard to malaria and identify key gaps that need to be addressed to improve readiness for malaria elimination. MERA expands on the typical HFA to include documentation of practices that malaria elimination experts at WHO and other groups have identified as necessary, such as ability to conduct high quality data reporting, data analysis on a local level, and clear roles and responsibilities in the event of an outbreak [18–23]. Furthermore, it includes perspectives of a range of providers and stakeholders at all levels of the system regarding barriers to high-quality service provision required for malaria elimination and opportunities for strengthening the malaria programme in elimination districts [24].

Malaria elimination programmes are expensive and labour intensive [12]. They require rigorous CM, testing all fevers in all ages throughout the year, treating all those positive with ACT, case investigation to determine whether the case was locally acquired, and reinforcing malaria knowledge and practices despite declining risk. MERA revealed that stock-outs of RDTs and appropriate treatment are relatively common in facilities and even more so among CHVs. Lack of trained staff, adequate storage space and irregular supervision visits were identified as key contributors to stock-out of malaria commodities. Hiring logisticians or other staff dedicated to commodity management at district level, clarifying the protocol for ordering routine and emergency stocks, and training HF staff and CHVs in that protocol were identified as key steps to improve commodity security.

In addition to reliable commodity stocks, their appropriate use is paramount to achieving malaria elimination. Most HPs and CHVs were able to identify that fever required testing with an RDT. However, nearly half of HPs did not obtain a fever history among patients who did not spontaneously report one, and nearly half of HPs did not record a temperature, which prevents accurate estimates of the number of suspected cases who receive an RDT. Fewer than half of patients with a fever complaint received an RDT, which is not adequate in an elimination programme that seeks to identify and treat every single case. A combination of approaches like training, supportive supervision, and other strategies such as group problem solving or quality improvement collaboratives that address systematic assessment of fever history and testing all fevers might be beneficial [25, 26]. Madagascar currently employs a multi-species RDT that differentiates between falciparum and general non-falciparum malaria, and outpatient facilities like those surveyed are not required to perform microscopy. Some HPs reported diagnosing *P. vivax* in their facilities, though it was not clear how they did so or what the protocol for testing is. Additional clarity in how to manage and report non-falciparum malaria, and *P. vivax* specifically, would help further address the malaria burden.

Another core recommendation for malaria elimination is to adapt surveillance systems to become more granular and quick to respond to unexpected increases in cases [21]. MERA illustrates how the health system is already partly prepared for elimination activities. Traditional malaria control programmes, including those in Madagascar, rely on passive surveillance with data reported monthly in aggregate. While adequate in high-transmission areas primarily focused on malaria control, an elimination programme must have a surveillance system that employs line listings of patients, including their location information and travel histories [18, 19]. HPs and CHVs reported asking for travel and location histories, and using that information in their decision-making, suggesting that it is possible to formalize the collection and use of this kind of information. Furthermore, asymptomatic cases must also be identified and treated as part of a strategy of infectious reservoir reduction. This can be done through active and reactive case detection [27–29]. Stakeholders note that a form of reactive case detection is already part of the response to outbreaks though there was little consensus on how that processes is triggered and who specifically is involved. Very few HPs reported participating in an outbreak response, and none of them recruited CHVs to assist them.

These recommended shifts in malaria surveillance for elimination require a change in data management and how data are used [20]. In Madagascar, data are abstracted from HF and CHV paper registers monthly, aggregated in a separate reporting form, and sent to the district, then region before joining the national dataset. At the national level, the data are analysed and interpreted with results sent down to the district and occasionally HF level. The cycle can take weeks to complete, thus preventing timely identification and response to infection clusters. Formal data analysis occurs at the central level and is not currently part of the role of those working in HFs. There are no guidelines instructing HPs on how to perform basic data analysis or what to prioritize. Despite this, HPs and CHVs noted using their data to guide decision-making, suggesting that this can be formalized, enabling faster response to outbreaks. Consensus at higher levels around epidemic thresholds, and training and supervision on thresholds at lower levels of the health system, is an opportunity to formalize data use among HFs and CHVs. Best practices in malaria elimination are to use near-real-time electronic data entry with elements of data interpretation shifted to the HF and community level where possible [21, 23, 30]. The NMCP is planning to introduce electronic data entry at the district level with a future goal of direct data entry in facilities using computer tablets, although current plans are for reporting aggregate numbers rather than line listings of cases. Stakeholders raised concerns, however, that staff would need significant training to use the technology, and that the number of available tablets might not be adequate for high quality data entry. Reliable internet connections will also be required for data transmission after offline data have been entered.

The overall needs identified through this assessment are costly, and as stakeholders noted, funding is often disrupted or unavailable for specific purposes. Comparing the malaria burden against the district and facility-level MERA scores can inform where to direct limited resources. District-level scores can illustrate broadly which districts are the farthest from achieving elimination without further assistance. In this survey, Antananarivo-Atsimondrano had the lowest score and the widest range of facility scores, from 30 to 61. Discussions with the district officers might clarify why some facilities are higher performing than others. The solution to improving performance is not necessarily the same for all of them. MERA shows that the highest performing facility in this district also had the highest scores in resource availability and very low CM scores, suggesting in this case that a lack of commodities might not be the primary driver of poor CM. It is possible that the malaria transmission is very low in this area such that HPs might benefit from targeted CM refresher training on testing all fevers. Of note, low disease burden might also explain why only one patient with a negative RDT was treated by ACT. The facility with the lowest overall score had a higher CM score with poor resource availability, suggesting that improving commodity management might help improve their performance.

Preliminary results of this MERA were used to draft the country’s elimination strategy and elimination surveillance plan. In addition, preliminary results were shared with stock management teams to facilitate discussions of improving quantification and management in districts targeted for elimination. Results will also be used for clarifying NMCP policies. For example, it was not clear why nearly a quarter of CHVs were no longer providing malaria testing and treatment services for children under 5 years of age in their catchment area. Historically, the focus of CHVs’ work was to provide services in communities situated greater than 5 km from a HF, including testing febrile children 2–59 months of age with an RDT, and treating those who were positive (and without signs of severe disease) with an ACT. The 2019–2030 Madagascar Community Health Strategy (*Strategie Nationale de Santé Communautaire*) does not include any geographic limitation for CHVs; however, some HF directors may limit the scope of work of their CHVs based on their needs or specific situations (e.g., limited supplies). This may be particularly true in areas with low malaria transmission such as elimination-targeted areas. MERA results revealed a need for policy and practice clarification. MERA results also highlighted gaps in surveillance that may be addressed as the country expands the use of the District Health Information Software-2 platform to integrate various systems currently in place, and to more efficiently capture community-level data. Finally, MERA results are being used to develop operational plans for piloting an elimination programme in two districts.

There are several limitations to this study. The purpose of the score was to establish a method to assess and compare elimination readiness among districts and facilities to help inform resource allocation and elimination plan development, rather than to measure associations between indicators and outcomes. Therefore, construct validity was not assessed and correlations between selected indicators were not investigated. In instances where there was limited variability between scores, the tool will have limited use; however, there was substantial variability in some areas that will allow planners to inform designs and decisions. This assessment was primarily focused on the public sector, with only one private facility included per district and no representation from other sources of care, such as private pharmacies, which would be critical to malaria elimination if they are a large source of care for patients seeking treatment [22]. As the NMCP begins elimination planning in earnest, it might be beneficial to include more perspectives from other care providers such as traditional healers and military hospitals, as there might be different types of resource and knowledge gaps among these populations [31]. Finally, some methods, which were based on ethical or resource considerations such as assuring patients were properly treated or directly observing providers, may have affected responses and behaviours.

## Conclusion

Malaria elimination planning is increasingly incorporated into national malaria management strategies. It is resource intensive and requires commodity security, strong CM, a more granular surveillance system, and flexible staff and systems that can identify and respond quickly to changes in local epidemiology. As Madagascar develops an elimination strategy, this health system readiness assessment in low-incidence districts provides a snapshot of the current resource and personnel capacity to implement critical elimination activities. The MERA offers a useful method for comparing various components of the health system along four domains and identifies areas that may benefit from additional resources or training. The approach was found to be informative and useful when documenting malaria elimination readiness across a health system. The tools can be adapted to fit the specific goals and processes of other contexts. The MERA can be repeated at regular intervals to monitor for performance improvement as elements of an elimination programme are introduced and adaptations are made to a country’s malaria programme.

## Supplementary information

**Additional file 1: Table S1.** All indicators that comprise the malaria elimination readiness domains for the MERA survey.

**Additional file 2:** MERA survey tool.

**Additional file 3:** MERA scoring tool by domain.

**Additional file 4: Table S2.** Malaria elimination readiness score and individual domain scores by facility system (out of 100).

## Data Availability

The datasets generated and/or analyzed during the current study have been de-identified and uploaded to USAID’s data development library (DDL).

## Abbreviations

ACT: Artemisinin-based combination therapy
ASAQ: Artesunate–amodiaquine
CM: Case Management
CHV: Community Health Volunteer
HF: Health Facility
HFA: Health Facility Assessment
HP: Health provider
MERA: Malaria Elimination Readiness Assessment
NGO: Non-Governmental Organization
NMCP: National Malaria Control Programme
SARA: Service Availability and Readiness Assessment
SOP: Standing Operating Procedure
RDT: Rapid diagnostic test
WHO: World Health Organization
