# Supplementary material for: Malaria case management and elimination readiness in health facilities of five districts of Madagascar in 2018
Source: Malar J. 2020 Oct 1;19:351. doi: 10.1186/s12936-020-03417-z (PMC7528237; doi:10.1186/s12936-020-03417-z)
Supplement: Supplementary file 1 — Additional file 1: Table S1. All indicators that comprise the malaria elimination readiness domains for the MERA survey. [file 12936_2020_3417_MOESM1_ESM.docx]

Additional Table 1. All indicators that comprise the malaria elimination readiness domains for the MERA survey

|  | **Source** | | | |
| --- | --- | --- | --- | --- |
|  | **Health Facility** | **Health Provider** | **Clinical Observation** | **Community Health Volunteer** |
| **Domain 1: Resource availability** | | | | |
| Diagnostic capacity | Malaria RDTs present | Able to test for *P. vivax* |  | Malaria RDTs present |
|  | Malaria RDTs out of stock in past 2 months | Diagnoses *P. vivax* in their facility |  | Malaria RDTs out of stock in past 3 months |
| Essential medicines | Oral ACT or Quinine present |  |  | Oral ACT present for children ≤ 5 years |
|  | Oral ACT or Quinine out of stock in past 2 months |  |  | Oral ACT out of stock in past 3 months |
|  | Injectable Artesunate or Quinine present |  |  |  |
|  | Injectable Artesunate or Quinine out of stock in past 2 months |  |  |  |
|  | Primaquine present |  |  |  |
| Other commodities | Thermometer available |  |  | Thermometer available |
|  | Infant weighing scale available |  |  |  |
|  | Stand-on weighing scale available |  |  |  |
| Guidelines | National Malaria Guidelines available |  |  |  |
| Stock management system | Stock management system present |  |  | Stock management system present |
| **Domain 2: Case Management** | | | | |
| Suspect case |  | Identifies criteria for testing for malaria | Fever history obtained | Identifies criteria for testing for malaria |
|  |  |  | Client's temperature was taken | Identifies need to assess fever |
| Test and Treat |  | Denies treating without RDT during periods of high workload | Patients with fever complaint received RDT | Identifies need to test fever with RDT |
| Management of uncomplicated malaria |  | Reports treating malaria with first line antimalarial |  | Reports treating malaria with first line antimalarial |
|  |  | Identifies treatment options for *P. vivax* |  | Follows-up with cases after initial consultation |
| Accessing high risk populations |  |  |  | Performs outreach to pregnant women to seek care |
|  |  |  |  | Performs outreach regarding travel and malaria risk |
|  |  |  |  | Considers travel history in febrile patients |
| Community engagement | Malaria social behavior change activities |  |  |  |
|  | Malaria education conducted |  |  | Malaria education conducted |
| **Domain 3: Data Management and Use** | | | | |
| Data reporting |  |  |  | CHV maintains a patient registry |
|  | CHV reports data to the HF |  |  | CHV submits a monthly summary report |
|  | Able to map cases by location |  |  |  |
| Data analysis | Reviews data monthly |  |  | Uses register to make decisions |
|  | Data analysis SOPs available |  |  | Submit a monthly summary report |
| Data quality | Malaria cases identified as confirmed or clinically diagnosed in forms |  |  |  |
|  | Patient can be traced/followed from one register to another |  |  |  |
|  | Check the quality of the information in register |  |  |  |
|  | Data quality assessment SOPs or guidelines available |  |  | Data quality assessment SOPs or guidelines available |
|  | Uses register to make decisions |  |  |  |
| Epidemic response | Provides a response to an unexpected increase in cases |  |  | Provides a response to an unexpected increase in cases |
| **Domain 4: Training, Supervision, and Technical assistance** | | | | |
| Training | Overall HF given specific training in a variety of malaria sub-topics | Provider received general malaria training |  | Received data management training |
|  |  | Provider received specific training in a variety of malaria sub-topics |  |  |
|  | Overall HF given data management training | Provider received malaria elimination training |  |  |
| Supervision | Received malaria case management supervision visit |  |  | Received any kind of supervision visit |
|  | Received malaria data quality supervision visit |  |  |  |
| Feedback | Received feedback on data reporting |  |  | Received feedback on data reporting |
|  |  |  |  | Received feedback on case management indicators |
| Technical assistance | Received assistance in case management, malaria in pregnancy, or data management |  |  | Received guidance in responding to an outbreak |
|  | Received guidance in responding to an outbreak |  |  |  |
|  | Provides case management assistance to CHV |  |  |  |
|  | Provides data management assistance to CHV |  |  |  |
