# Supplementary material for: Malaria case management and elimination readiness in health facilities of five districts of Madagascar in 2018
Source: Malar J. 2020 Oct 1;19:351. doi: 10.1186/s12936-020-03417-z (PMC7528237; doi:10.1186/s12936-020-03417-z)
Supplement: Supplementary file 2 — Additional file 2: MERA survey tool. [file 12936_2020_3417_MOESM2_ESM.docx]

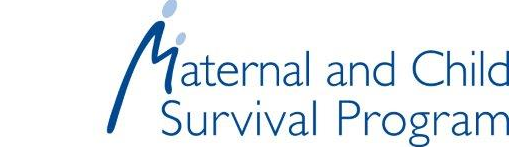


**ASSESSMENT TOOLS**

**Health Facility Assessment and Malaria Elimination Readiness Survey in Selected Districts of Madagascar**

**PI Version No. / Date: 29 Aug 2018**

# TOOL 1. HEALTH FACILITY CHECKLIST

**ALL HEALTH FACILITIES**

Note: Interviewers are asked to see the **Health Facility Manager** and ask to get the below information. In some cases, depending the reality in the field, the health facility manager may also be a health provider**.**

## **A. Health Facility Identification**

| **No.** | **Question** | | **Responses** | | |
| --- | --- | --- | --- | --- | --- |
| A01**: Date (DD/MM/YYYY)**  A02**: Interviewer’s name** | | |  | |  |
|  |  |  |  | |  |
| Health Facility Identification | | | | | |
| A03 | Type | | | (1)Public  (2)Private | |
| A04 | Health Facility Code | | |  | |
| A05 | Region | | | (note to programmer: insert regions here (will be a dropdown menu in the tablet) | |
| A06 | District | | | (note to programmer: insert districts here (will be a dropdown menu in the tablet) | |
| A07 | Health Facility Type* (picklist) | | | 1.Centre Hospitalier Universitaire (CHU)  2.Centre Hospitalier de Région (CHR)  3.Centre Hospitalier de District (CHD)  4.Centre de Santé de Base II (CSB II)  5.Centre de Santé de Base I (CSB I)  6.Autres (specifier) _____________________ | |
| A08 | Ambulatory only | | | 1.Yes  2.No | |
| A09 | Altitude |  | | | |
| A10 | Latitude |  | | | |
| A11 | Longitude |  | | | |
| A12 | About how many people live in this CSB coverage area? | \|_____\| people | | | |
| A13 | Is there a map of all households in the coverage area? | 1.Yes  2.No  99.Don’t know | | | |

A14. What is your highest degree/training?

(1)Professor

(2)Doctor

(3)Nurse

(4)Midwife

(5)CHW

(6)Other (Specify)____________

A15. What is your current position/role within this Health Facility?

(1)Healthcare Provider

(2)Manager

(3)Other (Specify) ____________

***The Health Facility in charge manager should be interviewed starting here.***

| **Health providers training** | | | | | | | | | | | | | |
| --- | --- | --- | --- | --- | --- | --- | --- | --- | --- | --- | --- | --- | --- |
| **Training and Services** | **Health provider type** | | | | | | | | | | | | |
|  | **Total** | **Physician Generalist  (non-specialist)** | **Certified nurse** | **Certified midwives** | **CHW** | **Specialist Gynecologist/obstetrician** | **Specialist Pediatrics** | **Pharmacist** | **Pharmacist Assistant** | **Laboratory Technician** | **Radiologist** | **Support staff** | **Other (specify)** |
| **B01** Total number of HF employees? |  |  |  |  |  |  |  |  |  |  |  |  |  |
| **B02**. Number who diagnose and treat malaria in their role |  |  |  |  |  |  |  |  |  |  |  |  |  |
| **B03.** Number who have received training on malaria in the last 2 years |  |  |  |  |  |  |  |  |  |  |  |  |  |

## **B. Human Resources at Health Facility level**

## **C. Existing care services**

| C01. Does this facility provide diagnosis and treatment of malaria? | 1.Yes | 2.No (go to Section D) |
| --- | --- | --- |
| C02. Which of the following methods are used in this facility to diagnose malaria? (**check all that apply**) | | |
| - Clinical symptoms | 1.Yes | 2.No |
| - Rapid Diagnostic Test | 1.Yes | 2.No |
| - Microscopy | 1.Yes | 2.No |
| C03.Do malaria service providers provide the following services to pregnant women as part of routine antenatal care services when stocks of commodities are available? (***check all that apply***) | | |
| - Iron supplementation | 1.Yes | 2.No |
| - Provision of ITNs at the first ANC visit | 1.Yes | 2.No |
| - RDT to diagnose malaria during pregnancy, if indicated | 1.Yes | 2.No |
| -Provision of intermittent preventive treatment in pregnancy (IPTp) with SP | 1.Yes | 2.No |

## **D. Availability of equipment and key tools**

| D01.Do you have the national malaria guidelines available in this facility today? IF YES, ASK TO SEE GUIDELINES | 1.Yes, observed  2.Yes, reported but not seen (skip to D03)  3.No (Skip to D03) | | | | | | |
| --- | --- | --- | --- | --- | --- | --- | --- |
| D02.If National Malaria Guidelines are seen, document the date of the guidelines. (DD/MM/YYYY) | _________________ | | | | | | |
| D03. For each item below, tell me if it’s available today and if it works. ASK TO SEE THE ITEM | **A) Available** | | | | **B) Functioning** | | |
|  | Observed | Reported but not seen | Not available | | Yes | No | Don’t know |
| a.Thermometer | 1 → B | 2 | 3 | | 1 | 2 | 8 |
| b.Infant weighing Scale | 1 → B | 2 | 3 | | 1 | 2 | 8 |
| c.Stand-on weighing scale | 1 → B | 2 | 3 | | 1 | 2 | 8 |
| D04.Does the health facility do microscopy? | 1.Yes  2.No (skip to question E1) | | | | | | |
| D05. If this facility has microscopy, what is an average number of malaria slides examined per week in your Health Facility? | 1.Less than 3  2.4 to 7  3.More than 8  4.do not know | | | | | | |
| D06. For each item below, tell me if it's available today and if it works. ASK TO SEE THE ITEM | **A) Available** | | | | **B) Functioning** | | |
|  | Observed | Reported but not seen | | Not available | yes | No | Don’t know |
| a.Staining supports | 1 → B | 2 | | 3 | 1 | 2 | 8 |
| b.Staining vessels | 1 → B | 2 | | 3 | 1 | 2 | 8 |
| c.Glass coloring dishes | 1 → B | 2 | | 3 | 1 | 2 | 8 |
| d.Drying slides | 1 → B | 2 | | 3 | 1 | 2 | 8 |
| e.Microscope | 1 → B | 2 | | 3 | 1 | 2 | 8 |
| f.Timers | 1 → B | 2 | | 3 | 1 | 2 | 8 |
| g.Pointing counters | 1 → B | 2 | | 3 | 1 | 2 | 8 |
| h.Differential counters | 1 → B | 2 | | 3 | 1 | 2 | 8 |
| i.Malaria blood slides | 1 → B | 2 | | 3 | 1 | 2 | 8 |
| j.Methanol | 1 → B | 2 | | 3 | 1 | 2 | 8 |
| k.Wright-Giemsa stain or other acceptable parasitic dye of malaria (e.g. Field A and B staining) | 1 → B | 2 | | 3 | 1 | 2 | 8 |
| l.Lab Coats / Aprons | 1 → B | 2 | | 3 | 1 | 2 | 8 |
| m.Disinfecting solution for lab cleaning | 1 → B | 2 | | 3 | 1 | 2 | 8 |
| n.Cotton and 70% alcohol | 1 → B | 2 | | 3 | 1 | 2 | 8 |
| o.Lancets | 1 → B | 2 | | 3 | 1 | 2 | 8 |
| p.Gloves | 1 → B | 2 | | 3 | 1 | 2 | 8 |
| q.Sharps container | 1 → B | 2 | | 3 | 1 | 2 | 8 |

**E. Availability of basic medicines**

| Are the following medications and RDTs in stock today? | In stock today (drug should be seen by data collector to be considered **in stock**)? |
| --- | --- |
| E01a. Artesunate-Amodiaquine (AS+AQ) (for < 1 year in age) | (1)Yes  (2)No…. GO TO E02a |
| E01b. Has artesunate amodiaquine (for < 1 year in age) been out of stock in the last 2 completed months? *For example, if the team visits the facility 7 August, this would include June and July.* | (1)YES  (2)NO…. GO TO E02a |
| E01c. How many days out of stock in last 2 months? | \| ______ # of days out of stock \|  \|  \| \| --- \| --- \| --- \| \|  \|  \|  \| \|  \|  \|  \| |
| E02a. Artesunate-Amodiaquine (AS+AQ) (for 1-4 years) | (1)Yes  (2)No…. GO TO E03a |
| E02b. Has artesunate amodiaquine (for 1-4 years) been out of stock in the last 2 completed months? *For example, if the team visits the facility 7 August, this would include June and July.* | (1)YES  (2)NO…. GO TO E03a |
| E02c. How many days out of stock in last 2 months? | \| ______ # of days out of stock \|  \|  \| \| --- \| --- \| --- \| \|  \|  \|  \| \|  \|  \|  \| |
| E03a. Artesunate-Amodiaquine (AS+AQ) (for 5-13 years) | (1)Yes  (2)No…. GO TO E04a |
| E03b. Has artesunate amodiaquine (for 5-13 years) been out of stock in the last 2 completed months? *For example, if the team visits the facility 7 August, this would include June and July.* | (1)YES  (2)NO…. GO TO E04a |
| E03c. How many days out of stock in last 2 months? | \| ______ # of days out of stock \|  \|  \| \| --- \| --- \| --- \| \|  \|  \|  \| \|  \|  \|  \| |
| E04a. Artesunate-Amodiaquine (AS+AQ) (for 14+ years) | 1)Yes  (2)No…. GO TO E05 |
| E04b. Has artesunate amodiaquine (for 14+ years) been out of stock in the last 2 completed months? *For example, if the team visits the facility 7 August, this would include June and July.* | (1)YES  (2)NO…. GO TO E05 |
| E04c. How many days out of stock in last 2 months? | \| ______ # of days out of stock \|  \|  \| \| --- \| --- \| --- \| \|  \|  \|  \| \|  \|  \|  \| |
| E05. Artemether-Lumefantrine (AL) | (1)Yes  (2)No |
| E06a. Artesunate (injectable) | (1)Yes  (2)No |
| E6b. Has Artesunate (injectable) been out of stock in the last 2 completed months? *For example, if the team visits the facility 7 August, this would include June and July.* | (1)YES  (2)NO…. GO TO E7 |
| E6c. How many days out of stock? | \| ______ # of days out of stock \|  \|  \| \| --- \| --- \| --- \| \|  \|  \|  \| \|  \|  \|  \| |
| E07a. SP (Sulfadoxine + Pyrimethamine) | (1)Yes  (2)No …. GO TO E08 |
| E07b. Has SP (Sulfadoxine + Pyrimethamine) been out of stock in the last 2 completed months? *For example, if the team visits the facility 7 August, this would include June and July.* | (1)YES  (2)NO…. GO TO E08 |
| E07c. How many days out of stock in last 2 months? | \| ______ # of days out of stock \|  \|  \| \| --- \| --- \| --- \| \|  \|  \|  \| \|  \|  \|  \| |
| E08a. Quinine (tablet) | (1)Yes  (2)No NO…. GO TO E9 |
| E08b. Has Quinine tablet been out of stock in the last 2 completed months? *For example, if the team visits the facility 7 August, this would include June and July.* | (1)YES  (2)NO…. GO TO E9 |
| E08c. How many days out of stock in last 2 months? | \| ______ # of days out of stock \|  \|  \| \| --- \| --- \| --- \| \|  \|  \|  \| \|  \|  \|  \| |
| E09a. Quinine (injectable) | (1)Yes  (2)No…. GO TO E10 |
| E09b. Has Quinine (injectable) been out of stock in the last 2 completed months? *For example, if the team visits the facility 7 August, this would include June and July.* | (1)YES  (2)NO…. GO TO E10 |
| E09c. How many days out of stock? | \| ______ # of days out of stock \|  \|  \| \| --- \| --- \| --- \| \|  \|  \|  \| \|  \|  \|  \| |
| E10. Chloroquine | (1)Yes  (2)No |
| E11. Primaquine | (1)Yes  (2)No |
| E12a. Malaria RDTs | (1)Yes  (2)No |
| E12b. Have RDTs been out of stock in the last 2 completed months? *For example, if the team visits the facility 7 August, this would include June and July.* | (1)YES  (2)NO |
| E12c. How many days out of stock in last 2 months? | \| ______ # of days out of stock \|  \|  \| \| --- \| --- \| --- \| \|  \|  \|  \| \|  \|  \|  \| |
| E13a. Insecticide-treated mosquito nets (ITNs) for ANC clinics and children < 5 with malaria | (1)Yes  (2)No |
| E13b. Have ITNs been out of stock in the last 2 completed months? *For example, if the team visits the facility 7 August, this would include June and July.* | (1)Yes  (2)No |
| E13c. How many days out of stock in last 2 months? | \| ______ # of days out of stock \|  \|  \| \| --- \| --- \| --- \| \|  \|  \|  \| \|  \|  \|  \| |
| E13d.Do you have a two-month supply of ITNs? | (1)Yes  (2)No |

## **F. Malaria Commodity Stock Management**

| F1 | Who provides you with malaria commodities?  *Do not read responses. Select all mentioned.* | 1.District office  2.Regional Office  3.DLP  4.Partners: ________  96.Others______________________ |
| --- | --- | --- |
| F2 | How often do you receive commodities?  *Do not read responses. Select all mentioned.* | 1.Weekly  2.Monthly  3.Quarterly  4.Twice a year  5.Annually  96.Other:_____________ |
| F3 | Are there expired RDTs in stock? (VERIFY by looking at dates on stock) | 1.Yes  2.No |
| F4 | Are there expired ACTs in stock? (VERIFY by looking at dates on stock) | 1.Yes  2.No |
| F5 | Do you have a two-month stock of RDT today? | 1.Yes  2.No  99.Don’t know |
| F6 | Do you have a two-month stock of ACT today? | 1.Yes  2.No  99.Don’t know |
| F7 | How do you request/ receive malaria commodities to be able to respond to an increase in cases?  *Do not read responses. Select all mentioned.* | 1.Request additional malaria commodities before the major transmission season  2.Request emergency supply of commodities once an outbreak has been detected  3.Request commodities from other HFs  4.Purchase commodities directly  96.Other(s): ___________________ |
| F8 | Is there a person assigned to lead or assist in managing malaria-related commodities at this HF? | 1.Yes; what is the position title for this person___________  2.No |
| F9 | Do you have a system for keeping track of your commodities in stock? | 1.Yes  2.No (skip to question F11) |
| F10 | If yes, how do you keep track of your commodities? | 1.Stock register  96.Other (specify) |
| F11 | Have **you** received **malaria commodity management** specific trainings in the last 2 years? | 1.Yes  2.No |

## **Social behavior change communication and community participation**

| **G1** | Do you have BCC activities specific to malaria in your community? | 1.Yes  2.No  99.Don’t know |
| --- | --- | --- |
| **G2** | What types of malaria education efforts are conducted by this HF?  *Do not read responses. Select all mentioned.* | 1.There are no educational efforts conducted  2.House to house health education  3.Community education  4.Printed health education materials  5.Health education at health facilities  6.Health education through health development army (1-5 network)  96.Other(s):____________________________ |
| **G3** | Does this Health Facility have a malaria prevention and treatment communication plan? | 1.Yes  2.No  99.Don’t know |
| **G4** | What topics/key information on malaria prevention and treatment do providers at this facility share with clients?  *Do not read responses. Select all mentioned.* | 1.Malaria diagnosis  2.Malaria prevention using ITNs  3.Name of the malaria medications  4.ANC and IPTp importance  5.Advising parents to return for follow-up care 2 days after children < 5 have been treated for malaria or sooner if their condition worsens.  6.For pregnant women receiving SP for IPTp, waiting 30 minutes after taking the dose before leaving  96.Others : specify |

## **H. Supervision**

| H1a | Did this facility receive any supervision visits in the last 6 months? | (1)Yes  (2)No ….GO TO I1  (99) Don’t know |
| --- | --- | --- |
| H1b | If yes, how many visits? | \| _____# of visits \|  \|  \| \| --- \| --- \| --- \| \|  \|  \|  \| \|  \|  \|  \| |
| H2 | Was malaria case management part of this supervision? | 1.Yes  2.No  99.Don’t know |
| H3 | Who did the supervision?  *Do not read responses. Select all mentioned.* | 1.National Staff  2.Regional Staff  3.District staff  4.Other (specify) ________________  99.Don’t know |
| H4 | What things related to malaria activities did your supervisor focus on during the visit?  *Do not read responses. Select all mentioned.* | 1.Commodity management (medications, RDTs, ITNs)  2.Vector control (IRS, ITN use)  3.Malaria reports/data  4.Lab supplies (RDT, slides, reagent, etc.)  5.Case management (diagnosis, treatment and follow-up)  6.Social and behavior change communication (SBCC)  96.Other:____________________ |
| H5 | During the last visit from a supervisor, did providers receive any feedback on their work? | 1.Yes (specify)___________  2.No |

## **Technical Assistance**

| **Technical assistance received by HF** | | |
| --- | --- | --- |
| I1 | Who does your HF regularly ask for technical assistance regarding malaria activities?  *Do not read responses. Select all mentioned.* | 1.This HF has not yet asked for technical assistance (Skip to question I5)  2.MOH  3.DLP  4.Regional health office  5.District health office  6. RPD  7. RPR  8.Partner(s) _____________­  96.Other(s):__________________________ |
| I2 | If the HF has not yet asked for technical assistance for malaria activities, what are the reasons?  *Do not read responses. Select all mentioned.* | 1.There was no need for technical assistance  2.We did not know how to request technical assistance  3.We are getting technical assistance without requesting for it  4.No case of malaria  96.Other(s):__________________________ |
| I3 | Please give me 1-2 examples of the type of technical assistance that was received?  *Do not read responses. Select all mentioned.* | 1.Training on the detection and management of malaria  2.Malaria in pregnancy  3.Malaria data management and reporting  96.Other _______________________ |
| I4 | What additional technical assistance does your HF need to reduce the burden of malaria?  *Do not read responses. Select all mentioned.* | 1.Training on the detection and management of malaria cases  2.Assistance in responding to outbreaks  3.Supportive supervision  4.Support on climate information  96.Other(s):_____________________ |

| **Technical assistance provided by HF to CHW** | | |
| --- | --- | --- |
| I5 | Does this HF provide malaria-specific technical assistance to the community health workers (CHWs) it supports? | 1.Yes  2.No (skip to question I7) |
| I6 | Please give me 1-2 examples on the types of technical assistance this HF has provided to CHWs?  *Do not read responses. Select all that apply.* | 1.Malaria diagnosis  2.Referral  3.Formative supervision  4.Data Management and Reporting |
| I7 | If it does not, what are the reasons this HF does not provide technical assistance to CHWs?  *Do not read responses. Select all mentioned.* | 1.No training in how to give technical assistance  2.Transportation problems  3.Budget shortage (per diem and other costs)  4.Not enough time or staff to give technical assistance  96.Other(s):____________________ |
| I8 | What are the challenges in collaborating with CHW?  *Do not read responses. Select all mentioned.* | 1.Lack of motivation of the CHW  2.Lack of reporting by the CHW  3.Delay in response from the CHW  4.Quality of services provided by the CHW  96.Other (specify)____________ |

## **Epidemic response**

| **EPIDEMIC RESPONSE** | | |
| --- | --- | --- |
| **J1** | Are you aware of the malaria outbreak threshold for your area? | 1.Yes  2.No  99.Don’t know |
| **J2** | Do you have a system for monitoring for malaria outbreaks in your area? | 1.Yes  2.No  99.Don’t know |
| **J3** | How does this facility respond to an increase in malaria cases?  *Do not read responses. Select all mentioned.* | 1.This HF does not have any capacity to coordinate responding to increase in malaria cases  2.Keep enough commodities to respond to 2 times or more times the usual malaria cases for this HF  3.Make emergency requests for malaria commodities from the District  4.Request assistance from the District  5.Refer severe malaria cases to other HFs/ hospitals for advanced care  6.Distribute additional ITNs in affected areas  7.Conduct active case detection/community-level outreach to test and treat cases in affected areas  8.Contact partners and request for assistance  9.Contact CHWs to conduct additional activities  96. Other(s):______________________________ |
| **J4** | Has this HF received any guidance on how to immediately respond to a malaria outbreak? | 1.Yes, who provided this guidance (MOH or partner)______  2.No  99.Don’t know |
| **J5** | Has this HF responded to an outbreak in the past two years? | 1.Yes  2.No (skip to question J7)  99.Don’t know |
| **J6** | What are the challenges when responding to outbreaks?  *Do not read responses. Select all mentioned.* | 1.There are no challenges when responding to outbreaks  2.No document or tools to guide systematic responses  3.Households are hard to reach  4.Community members have limited involvement in environmental management  5.People with fever do not come to the HF  6.Difficulty maintaining RDTs and ACTs stock  7.Affected community members are resistant to outreach efforts  8.Health workers/staff are not sure what action(s) to take  9.Health workers do not have time/capacity to conduct additional activities/services related to outbreaks.  96.Other(s):_________________ |
| **J7** | What is needed to improve the current malaria surveillance system(s)?  *Do not read responses. Select all mentioned.* | 1.Additional trainings  2.Budget support  3.Improve data recording  4.Provide reporting forms  5.Provide recording books  6.Provide data analysis tools (epidemic monitoring chart)  7.Additional human resources  8.Strengthening health education  9.Supportive supervision  96.Other(s): _____________________________ |

1. **SURVEILLANCE, MONITORING AND EVALUATION (SM&E)**

| **DATA MANAGEMENT AND DATA USE** | | | | | | | | |
| --- | --- | --- | --- | --- | --- | --- | --- | --- |
| K1 | Has anyone in this facility received training in recording, processing, or reporting of health information (**data management**) in the last 2 years? | 1.Yes  2.No (skip to question K3) | | | | | | |
| K2 | Please answer the following based on the **last** data management and use training  **Check all that apply and answer corresponding columns** | Subject of training | | Month and year | | Who provided the training? (MOH or partner – include name of partner) | | |
|  |  | 1.Data collection/ recording/ reporting | | 1a. | | 1b. | | |
|  |  | 2.Data displays | | 2a. | | 2b. | | |
|  |  | 3.Data analysis | | 3a. | | 3b. | | |
|  |  | 4.Other: | | 4a. | | 4b. | | |
| K3 | How does this facility report HMIS/routine data?  *Do not read responses. Select all mentioned.* | 1.HF staff complete RMA paper form  2.HF staff send RMA form to the district office  3.Someone from the district comes to fill out a paper RMA form  4.HF staff reports RMA data via SMS to the district  5.HF staff reports RMA data over the phone to the district officer  6.HF staff reports RMA data via tablet  96.Other:________________ | | | | | | |
| K4 | How does this facility report IDSR data?  *Do not read responses. Select all mentioned.* | 1.HF staff completes IDSR paper form  2.HF staff sends the IDSR paper form to the district office  3.Someone from the district comes to fill out a IDSR paper form  4.HF staff sends IDSR reports using SMS  5.HF staff sends IDSR data via tablet  6.HF staff sends IDSR data over the phone to the district officer  96.Other:________________ | | | | | | |
| K5 | Do you keep track of referrals from your CHWs? | 1.Yes  2.No  99.Don’t know | | | | | | |
| K6 | How often do you report routine data? | 1.Weekly  2.Monthly  3.Quarterly  4.Twice a year  5.Annually  96.Other: _____________ | | | | | | |
| K7 | Does this health facility have the ability to map cases from reports? | 1.Yes  2.No  99.Don’t know | | | | | | |
| K8 | Are there additional reporting requirements for when the outbreak threshold is exceeded? | 1.Yes  2.No (skip to question K11)  99.Don’t know | | | | | | |
| K9 | What indicators are included in the reporting requirements?  *Do not read responses. Select all mentioned.* | 1.Number of all consults  2.Number of consults with fever  3.Number of consults tested with RDT or microscopy  4.Number of consults with positive RDTs or microscopy  5.Number of consults treated with ACT  6. Number of consults followed-up for completion of treatment  7.Number of RDTs currently in stock  8.Number of ACTs currently in stock  9.Number of cases of severe malaria  10.Number of deaths due to malaria  11.Number of cases of malaria who traveled outside of the fokontany  96.Other: _______________ | | | | | | |
| K10 | In what time period are the outbreak reports due? | 1.Immediately after completion  2.With the next IDSR/weekly report  3.With the next monthly report  4. Other : ________  99.Don’t know | | | | | | |
| K11 | Do CHWs regularly submit their data for submission to the district? | 1.Yes  2.No (skip to K14)  99.Do not know | | | | | | |
| K12 | If yes, how often do you receive data from the CHWs? | 1.Weekly  2.Monthly  3.Quarterly  4.Twice a year  5.Annually  96.Other: _____________ | | | | | | |
| K13 | *If yes*, what proportion of the CHWs submit their report each month, on average? | 1.All CHWs  2.Not all but more than half the CHWs  3.About half the CHWs  4.Some but less than half  5.Only a few CHWs  99.Do not know | | | | | | |
| K14 | Does the HF receive feedback on the data reported to the district? | 1.Yes  2.No (skip to question K16)  3.Sometimes  99.Don’t know | | | | | | |
| K15 | In the past 3 months, did you receive any feedback on **malaria case management** indicators? |  | 1a. Verbal | | 1b. Written | | 1c. Phone call | 1d. Other: |
|  |  | 1.Yes |  | |  | |  |  |
|  |  | 2.No | | | | | | |
| K16 | What are some improvements, if any, you would like to see with the current malaria case reporting systems?  *Do not read responses. Select all mentioned.* | 1.No improvement is needed  2.Make the reporting form shorter  3.Collect information more often  4.Collect information less often  5.CSB should send someone to fill out the form  6.Support electronic data collection  7.Feedback on reported data should be given  96.Other: _______________________ | | | | | | |
| **DATA ANALYSIS** | | | | | | | | |
| K17 | Does this HF compare changes in malaria indicators routinely (data analysis)? (ex: changes in malaria cases from March to April) | 1.Yes  2.No (skip to question K19)  99.Don’t know | | | | | | |
| K18 | How often is malaria data analysis done?  ***(Tick all report types that apply for each time period selected by the respondent)*** | 1.Weekly  2.Every 2 weeks  3.Monthly  4.Quarterly  5.Twice a year  6.Annually  96.Other: _____________ | | | | | | |
| K19 | Do you have any SOPs/guidelines for analyzing your data? | 1.Yes  2.No  99.Don’t know | | | | | | |
| K20 | Are malaria cases identified as laboratory-confirmed or clinically diagnosed in forms used for tracking? | 1.Yes  2.No  99.Do not know | | | | | | |
| K21 | Can a patient be traced/followed from one register to another (i.e. from inpatient register to laboratory register to pharmacy register)?  ***Note to the surveyor****: investigate to make sure this process is / is not possible*. | 1.Yes, patient can be traced across **all** registers  If yes, by what mechanism:  1a.unique ID  1b.patient name  1c.other________________________  2.Yes, patient can be traced across **some** registers  If yes, by what mechanism:  2a.unique ID  2b.patient name  2c.other________________________  3.No  99.Don’t know | | | | | | |
| K22 | Do you check the quality of the information in your register? | 1.Yes (go to question K24)  2.No  3.Sometimes  99.Don’t know (go to question K24) | | | | | | |
| K23 | If no, why not?  *Do not read responses. Select all mentioned.* | 1.Have not received training  2.Don’t know how to do it  3.Did not think to do it  4. No time to do this  5.It is not necessary to check the register  6. Other (Specify):______________________ | | | | | | |
| K24 | Do you have any SOPs/guidelines for checking the quality of the information in your register? | 1.Yes  2.No  99.Don’t know | | | | | | |
| K25 | Has this facility been visited as part of a data quality audit in the past year? | 1a.Yes.  1b.Organization that performed the DQA ________________  2.No  99.Don’t know | | | | | | |
| K26 | Does your HF use malaria data to make evidence-based decisions?  (e.g.: if malaria data completeness is low one month, using this information to focus on improving malaria data completeness for the following month) | 1.Yes  2.No (skip to question K29)  99.Don’t know | | | | | | |
| K27 | If yes, when is malaria data used to make decisions?  *Do not read responses. Select all mentioned.* | 1.High case spikes  2.Low or stock out of malaria commodities  3.Malaria data is not used to make decisions  96.Other(s) (specify): __________________ | | | | | | |
| K28 | If yes, what types of decisions has this HF made with this data?  *Do not read responses. Select all mentioned.* | 1.Examining performance by looking at target goals and actual performance from month to month  2.Mobilizing/shifting of resources based on needs of the HF  3.Asking for more malaria support by showing gaps in ability to meet target (example: by requesting for more commodities)  96.Others (specify): ___________ | | | | | | |
| K29 | What are the reasons this HF doesn’t use malaria data to make decisions?  *Do not read responses. Select all mentioned.* | 1.Data is not analyzed  2.Do not know how to use it  3.Decisions are made at higher level  96.Other(s) (specify): _________________ | | | | | | |
| K30 | Does this HF have routine meetings where malaria-based information is discussed? | 1.Yes  2.No (skip to question K32)  99.Don’t know | | | | | | |
| K31 | In the past three months, what malaria information was discussed at the meetings?  *Do not read responses. Select all mentioned.* | 1.There was no malaria information discussed in the past 3 months  2.Managing malaria-related data regarding quality, reporting or timeliness of reports  3.Malaria commodity stock out and coverage of malaria interventions  4.Following up on malaria specific decisions and actions from previous meeting  5.Any malaria-specific (cases or commodity) issues or problems referred to higher levels  96.Other (specify) | | | | | | |
| K32 | Has this HF experienced any challenges with managing and using malaria data? | 1.Yes  2.No (skip to question L1)  99.Don’t know (skip to question L1) | | | | | | |
| K33 | Please give me one or two examples of this? |  | | | | | | |

1. **HUMAN RESOURCE CAPACITY**

| L1 | How many CHWs are associated with this facility? | ____ # of CHW |
| --- | --- | --- |

| L2 | Are the staff at this HF able to meet the needs of this community for malaria activities? | 1.They meet them very well (skip to question L4)  2.They meet them to some extent  3.They don’t meet them at all |
| --- | --- | --- |
| L3 | What are some of the reasons why health workers are not able to meet the needs of this community for malaria activities?  *Do not read responses. Select all mentioned.* | 1.Work overload  2.High turnover of health workers  3.Could not fill vacancy  4.Not having enough malaria commodities  5.Lack of well-trained health workers  6.Difficult landscape/topography of the area  7.Long distance to community members  8.Budget shortage (fuel, transportation to HF, to make supervision visits, etc.)  96.Other(s): _____________ |
| L4 | How has staff turnover been in this HF? | 1.Major problem  2.Minor problem  3.Not a problem  99.Do not know |
| L5 | What is the number of HF staff that have left in the past 12 months? | *\|____\|* |
| L6 | How many of those positions have been filled? | 1.All of them  2.Some of them  3.None |
| L7 | What are the reasons for staff turnover?  *Do not read responses. Select all mentioned.* | 1.Turnover is not a problem in this HF  2.No opportunities for promotions  3.Transitioning to other positions within HF  4.Leaving for opportunities outside of healthcare  5.No appropriate housing near the HF Poor transportation to HF  6.Long distance to the HF  7.Low salaries  8.Lack of supervision/support  96.Other(s):_______________ |

***Note to data collector: this is for elimination districts only***

| L8 | Does this facility have enough staff currently to undertake malaria elimination activities? | 1.Yes  2.No  99.Don’t know |
| --- | --- | --- |
| L9 | If “No”, how many additional staff, including CHWs does this facility need? | \| Position Title \| # of staff \| \| --- \| --- \| \| Clinical Staff \| *\|__\|__\|* \| \| Lab Staff \| *\|__\|__\|* \| \| Pharmacy Staff \| *\|__\|__\|* \| \| HMIS Focal Person \| *\|__\|__\|* \| \| CHWs \| *\|__\|__\|* \| |

# TOOL 2. PROVIDER HEALTH FACILITY INTERVIEW GUIDE

## **Profile**

**Code:**

| A1.Participant code | *Region, District, sub-district, participant category, specific participant Code (additional information on how to collect this code will be attached to this guide)* | |
| --- | --- | --- |
| A2.Qualification | 1.Doctor  2.Nurse  3.Midwife  96.Other (specify) _____________ | |
| A3.Years in current position | *_____ # of years in current position* |  |

## **Staffing availability and training**

| **STAFF TRAINING** | | | | | |
| --- | --- | --- | --- | --- | --- |
| B1 | Have you received any malaria-specific trainings in the last 2 years? | 1.Yes  2.No (go to question B3)  99.Don’t know | | | |
| B2 | What was the content of these trainings?  *Do not read responses. Select all mentioned.* | 1. Malaria diagnosis/use of RDTs/microscopy  2.Fever management  3.Treatment for uncomplicated malaria  4.Treatment for severe malaria  5.Malaria in pregnancy  6.Epidemic thresholds  7.Malaria Elimination  8.Malaria surveillance and monitoring  96.Other (specify): ______________________ | | | |
| B3 | What additional malaria trainings do you think would be helpful for you or your staff to have?  *Do not read responses. Select all mentioned.* | 1.No additional trainings will be useful  2.Malaria diagnosis  3.Malaria treatment  4.Management of severe and complicated malaria  5.Management of non-malaria febrile illness  6.M&E of malaria  7.Malaria in pregnancy/IPTp  8.Integrated Community Case Management (iCCM)  9.Social & Behavior Change Communication (SBCC) and malaria  10.Epidemic detection and response  96.Other(s)_________________ | | | |
| B4 | What are some of the challenges providers have experienced using the knowledge gained from the malaria trainings received?  *Do not read responses. Select all mentioned.* | 1.There have been no challenges experienced  2.Not having enough trainings  3.The topics covered during the training do not help with work  4.Malaria supplies/commodities not available at facility  5.High staff turnover  6.No coverage when provider is absent or in training  96.Other(s):___________________ | | | |
| B5 | Have you received training in malaria monitoring and evaluation? | 1.Yes  2.No (go to question B8) | | | |
| B6 | On the **last** malaria monitoring and evaluation specific trainings **you** received, please provide the details**?**  **Check all that apply and answer corresponding columns** | Number of trainings on **monitoring and evaluation you** received within the last 2 years. | | | *\|__\|__\|* |
|  |  | Subject of training | Month and year | Who provided the training?  □ MOH  □ Partner – include name of partner) | Was it useful to your work?  □ Yes  □ No |
|  |  | 1.HMIS (RMA) |  |  |  |
|  |  | 2.IDSR( *weekly integrated disease surveillance and reporting system)* |  |  |  |
|  |  | 3.Tracking cases |  |  |  |
|  |  | 4.Epidemic Threshold |  |  |  |
|  |  | 5.Electronic-based (tablet) reporting |  |  |  |
|  |  | 6.Other |  |  |  |
| B7 | What are some of the challenges providers experience using the knowledge gained from the malaria **monitoring and evaluation training** training you received?  *Do not read responses. Select all mentioned.* | 1.There were no challenges experienced  2.Not having enough trainings  3.The topics covered during the training do not help with my work  96.Other(s)___________________ | | | |
| B8 | What additional malaria M&E trainings would providers like to receive?  *Do not read responses. Select all mentioned.* | 1.No additional trainings will be useful  2.HMIS  3.IDSR  4.Epidemic thresholds  5.Case-based reporting  6.Active case detection  7.Analysis and use of malaria data Outbreak response  96.Other(s)_________________ | | | |

## **C.Malaria Case Management**

| **Testing and Diagnosis** | | |
| --- | --- | --- |
| C1 | How does one decide if a patient is a suspect case of malaria?  *Do not read responses. Select all mentioned.* | 1.Patient reports history of fever at home  2.Patient has fever during visit  3.Age of patient  4.febrile patient reports no fever but reports at least one of the following symptoms (chills, sweats, headache, body aches, nausea, vomiting, abdominal pain, diarrhea)  5.Patient presents to clinic for any sign/symptom and reports travel to high transmission region  6.Based on time of year/Symptomatic patient presents during transmission season  96.Other: _____________________ |
| C2 | If a RDT is used, what is the reason for using RDT, rather than microscopy? | 1.No laboratory  2.No microscopist  3.No functioning microscope  4.Lack of reagent  5.Supply shortage  6.Power supply interruption  96.Other____________________________ |
| C3 | If the workload of this HF is very heavy, do you skip the RDT and treat the patient without testing? | 1a.Yes  1b.If yes, reason: _____________________  2.No |
| C4 | Are providers able to test for *P. vivax*? | 1.Yes  2.No  99.Don’t know |
| C5 | Are providers able to test for G6PD deficiency? | 1.Yes  2.No  99.Don’t know |
| C6 | Do providers have treatment guidelines for *P. vivax*? (ask to see) | 1a.Yes, unverified  1b.Yes, verified  2.No  99.Don’t know |
| C7 | Do providers receive referrals for severe malaria suspected cases from CHWs? | 1.Yes  2.No  99.Don’t know |
| C8 | What clinical signs or symptoms would suggest to you that a patient needs referral (or admission) for severe malaria?  *Do not read responses. Select all mentioned.* | 1.Convulsions  2.Altered consciousness  3.Abnormal breathing  4.Vomiting  5.Diarrhea  96.Other:_________________ |
| C9 | Once one has determined the patient has severe malaria, what should be done?  *Do not read responses. Select all mentioned.* | 1.Administer injectable artesunate  2.Administer IV fluids  3.Refer to the hospital  4.Administer rectal artesunate  96.Other:_________________ |
| C10 | What are the challenges providers experience with diagnosing malaria cases?  *Do not read responses. Select all mentioned.* | 1.There are no challenges with diagnosing malaria cases  2.RDT (or microscopy supply)stock outs  3.Lack of training  96.Other(s):_______________ |
| **Malaria Treatment Activities** | | |
| C11 | What type of malaria treatment options do providers at this HF provide for uncomplicated ***P. falciparum***?  *Do not read responses. Select all mentioned.* | 1.Artesunate-Amodiaquine (AS+AQ)  2.Artemether-Lumefantrine  3.Artesunate (injectable)  4.Artesunate (rectal suppository)  5.Quinine (tablet)  6.Quinine (injectable)  7.Chloroquine  8.Primaquine  96.Other(s):____________ |
| C12 | If AS+AQ is not given, why not? | 1.Artesunate-Amodiaquine (AS+AQ) is not available  2.Artesunate-Amodiaquine (AS+AQ) is not effective  3.Artesunate-Amodiaquine (AS+AQ) is too expensive  96.Other(s):____________ |
| C13 | Do you diagnose ***P. vivax*** in your HF? | 1.Yes  2.No (go to question C15) |
| C14 | What type of malaria treatments does this HF provide for ***P. vivax***?  *Do not read responses. Select all mentioned.* | 1.Artesunate-Amodiaquine (AS+AQ)  2.Artemether-Lumefantrine  3.Artesunate (injectable)  4.Artesunate (rectal suppository)  5.Quinine (tablet)  6.Quinine (injectable)  7.Chloroquine  8.Primaquine  96.Other(s):____________ |
| C15 | If AS+AQ & Primaquine are not given, why not? *Do not read responses. Select all mentioned.* | 1.ASAQ & Primaquine is not available  2.ASAQ & Primaquine is not effective  3.ASAQ & Primaquine is too expensive  4.Primaquine only  5.Primaquine is not available  6.Primaquine is too expensive  7.ASAQ is not available  8.ASAQ is too expensive  96.Other(s):____________ |
| C16 | Which drugs do providers use for treatment of severe malaria cases in your setting?  *Do not read responses. Select all mentioned.* | 1.Do not treat severe malaria; only refer  2.ACT tablets  3.Quinine injection  4.Artesunate injection  5.Rectal artesunate  6.Quinine tablets |
| C17 | What challenges has this HF experienced with treating malaria cases?  *Do not read responses. Select all mentioned.* | 1.There no challenges with treating malaria cases  2.Medication stock out  3.Lack of staff training  4.Lack of treatment adherence (people do not take full treatment of ACTs)  5.Patients share prescribed antimalarial medication (with family member and others)  6.Clients lack funds for transport when referred  7.Clients lack funds to pay for treatment/hospitalization when referred  8.People seek treatment with traditional healers  9.People seek treatment late at the facility  10.Power shortage  11.Clean water shortage  96.Other(s):____________ |
| C18 | Is there follow-up on each malaria case to ensure treatment was completed and to address potential complications and side effects? | 1.Yes  2.No  99.Don’t know |

***Note to data collector : this is for elimination districts only***

| C19 | Have you received malaria elimination specific trainings? | 1.Yes  2.No (End of this tool) |
| --- | --- | --- |
| C20 | If “Yes”, what elimination activities were discussed during training?  *Do not read responses. Select all mentioned.* | 1.Case-based reporting  2.Active case detection  3.Geo-tagged reporting  4.Use of malaria data for decision-making  5.Epidemic thresholds and response  6.Fever testing policy  7.Special populations  96.Other: _________________ |

# TOOL 3: OBSERVATION GUIDE FOR HEALTH PROVIDER

Please observe the health care provider consulting with the patient without providing verbal or other comments and complete the answers to the following questions

| A1 | Did the patient report fever or history of fever in last 24 hours during consent process? | 1.Yes  2.No |
| --- | --- | --- |
| A2 | Did the provider welcome the patient? | 1.Yes  2.No |
| A3 | Did the provider invite the patient to sit down? | 1.Yes  2.No |
| A4 | Did the provider ask questions to the patient about his/her reasons for consultation? | 1.Yes  2.No |
| A5 | What is the patient’s age? | 1. <5 years  2. 5+ years |
| A6 | Does the patient complain of fever? | 1.Yes  2.No |
| A7 | What else does the patient complain of?  ***Check all that apply.*** | 1.Cough  2.Runny nose  3.Headache  4.Chills  5.Stomach ache  6.Loss of appetite  7.Diarrhea  8.Seizures  9.Abnormal breathing  10.Vomiting  11.Diarrhea  12.Body pain  96.Other (specify) :_____________ |
| A8 | If the patient did not complain of a recent fever, did the provider ask about recent or current fever ? | 1.Yes  2.No |
| A9. | *If child <5 years of age has or reports cough or abnormal breathing,* was respiratory rate measured? | 1.Yes  2.No  88.NA |
| A10 | Was the client’s temperature taken? | 1.Yes  2.No (go to A15) |
| A11 | If yes, was it taken correctly? | 1.Yes  2.No |
| A12 | Has the temperature been read? | 1.Yes  2.No |
| A13 | Was the temperature recorded in the register? | 1.Yes  2.No |
| A14 | If yes, what was the patient’s temperature? | ___ ___ . ___ ⁰C |
| A15 | Was a malaria rapid diagnostic test (RDT) done? | 1.Yes  2.No (go to A21) |
| A16 | If yes, was the test positive or negative? | 1.Positive  2.Negative |
| A17 | Has the result of the RDT been shared with the patient? | 1.Yes  2.No |
| A18 | Was a microscopy test performed? | 1.Yes  2.No (go to A21) |
| A19 | If yes, was the test positive or negative? | 1.Positive  2.Negative |
| A20 | Has the result of the microscopy test been shared with the patient? | 1.Yes  2.No |
| A21 | For a female patient between 12-49 years old, did the provider assess for pregnancy ? | 1.Yes  2.No |
| A22 | Was a test for anemia done? | 1.Yes  2.No  88.NA |
| A23 | If the result of the RDT or microscopy is negative, does the provider seek other cause of the fever? | 1.Yes  2.No  88.NA |
| A24 | What diagnosis/es was given to the patient?  *Check all that apply* | 1.No diagnosis given  2.Malaria  3.Diarrhea  4.Cough/cold  5.Acute respiratory infection  6.Pneumonia  7.Allergy  8.Ear infection  9.Skin problem/rash  10.Injury  96.Other (specify):______________ |
| A25 | Was the patient clearly told the diagnosis? | 1.Yes  2.No |
| A26 | What treatment did the health provider prescribe to the patient?  *Check all that apply.* | **Antimalarials**  1.ASAQ  2.AL  3.Quinine tablets  4.Quinine injection  5.SP  6.Chloroquine  7.Other antimalarial (specify) : _______  **Antibiotics**  8.Amoxicillin  9.Erythromycin  10.Cotrimoxizole  11.Other antibiotic (specify) :  **Other**  12.Oral rehydration solution  13.Deworming medication  14.Iron  15.Other (specify) :__________ |
| A27 | Have instructions been given to the patient for follow-up care? | 1.Yes  2.No |
| A28 | Was the patient reminded to sleep under an ITN? | 1.Yes  2.No |
| A29 | Did the health provider use a checklist during the encounter? | 1.Yes  2.No |
| A30 | Was the patient told when or under what circumstances (e.g., if symptoms do not resolve) to return to the health facility? | 1.Yes  2.No |
| A31 | What was the drug dispensed to the patient? (either with the provider or at the pharmacy) | 1.Yes  2.No |
| A32 | Was the patient counseled on how to take the medication? (either with the provider or at the pharmacy) | 1.Yes  2.No |

# TOOL 4: KNOWLEDGE TEST QUESTIONNAIRE FOR HEALTH PROVIDER

Instructions: please note that for some questions, more than one answer can be selected. Those questions have been marked as such. If you have any questions while you are filling this out, please contact a study team member.

| **GENERAL QUESTIONS** | | |
| --- | --- | --- |
| A01. | Malaria is transmitted by the bite of all kinds of mosquitoes | 1.True  2.False |
| A02. | The mosquito that transmits malaria bites mainly at night | 1.True  2.False |
| A03. | Fever and malaria are the same thing | 1.True  2.False |
| A04. | As soon as there is fever, there is suspicion of malaria | 1.True  2.False |
| A05. | The best way for your patients to prevent malaria is to sleep under a mosquito net impregnated with Insecticide | 1.True  2.False |
| A06. | List symptoms of uncomplicated malaria  (select all that apply) | 1.Fever or history of fever  2.Convulsion  3.Vomiting  4.Fatigue |
| A07. | How can a case of malaria be confirmed?  (select all that apply) | 1. Do an RDT  2. Do a thick or thin blood film  3. You do not have to confirm if it is malaria with a test; if there is a fever, the person should be treated for malaria  4. Only a and b are correct |
| A08. | An RDT or microscopy is needed to determine if a patient is suffering from malaria | 1.True  2.False |
| A09. | If a patient reports fever at home, but does not have fever at the clinic, they do not need testing | 1.True  2.False |
| A10. | If a patient presents with a fever, he or she should be tested with a malaria RDT or microscopy before being treated for malaria | 1.True  2.False |
| A11. | What is the first-line treatment for uncomplicated malaria in patients who are in their second or third trimesters of pregnancy?  (select all that apply) | 1.AS+AQ  2.SP  3.AL  4.Chloroquine  5.Fansidar  6.Quinine tablet  7.Quinine injectable  8.Artesunate injectable  9.Artesunate rectal  96.Other |
| A12 | If the result of RDT is negative, what are the next steps?  (select all that apply) | 1.Evaluate for respiratory infection  2.Evaluate for diarrheal disease  3. Evaluate for dehydration  4.Evaluate for pregnancy as needed  5.Evaluate for other cause of fever |
| A13 | The following signs may indicate the severity of malaria:  (select all that apply) | 1. Seizures  2.Fever greater than 40 °  3.Jaundice  4.Repeated vomiting  5..Hypertension  6.Unconsciousness  7.Deep breathing  8.Black coloring on the face  9.Breathing difficulties  10.Abnormal spontaneous bleeding |
| A14 | A high fever associated with one or more signs of severe malaria should lead to RDT to confirm malaria | 1.True  2.False |
| A15 | In addition to malaria, cite the other causes of fever that you would think of in a child under 5 years of age?  (select all that apply) | 1.Respiratory infection  2.Diarrheal disease  3.Vaccination  4.Accident/injury  5.Sepsis  96.Other |
| A16 | In addition to malaria, cite the other causes of fever that you would think of in adolescents and adults?  (select all that apply) | 1.Respiratory infection  2.Diarrheal disease  3.Accident/injury  4.Sepsis  96.Other |
| A17 | What is the first-line treatment for malaria in a pregnant woman in her first trimester? | 1.AS +AQ  2.SP  3.AL  4.Chloroquine  5.Fansidar  6.Quinine tablet  7.Quinine injectable  8..Artesunate injectable  9.Artesunate rectal  96.Other |
| A18 | Which key information is needed in terms of results of the microscopic examinations? | 1.Positivity only  2.Parasite density  3.Positivity and parasite density |
| A19 | A 4-year-old child consults for a fever. *See questions below* | |
| A19a | Do you need to order laboratory tests for this patient? | 1.Yes  2.No |
| A19b | What tests would you order? | 1.Microscopy (go to A20)  2.RDT  3.Others (specify):___________(go to A20) |
| A19c | How many drops of buffer solution do you use for an RDT? |  |
| A19d | How many minutes do you wait before reading the test? |  |
| A20 | How would you decide if a patient had been cured of malaria? | 1.No more temperature  2.RDT test negative  3.Microscopy test negative  99.Do not know |

# TOOL 5: INTERVIEW GUIDE WITH THE COMMUNITY HEALTH WORKERS– *at Elimination Districts only*

## **Community Health Worker Site Identification and Description**

| **No.** | | **Question** | **Responses** | |
| --- | --- | --- | --- | --- |
| **A01 Date (DD/MM/YYYY)**  **A02 Interviewer’s name** | |  |  | |
|  |  |  |  | |
| CHW Site Identification | | | | |
| **A03** | Name of Community | | |  |
| **A04** | Code of affiliated CSB | | |  |

| A05.CHW Participant code | *Region, District, specific participant number (additional information on how to collect this code will be attached to this guide)* |
| --- | --- |
| A06.Education | 0: No education / 1: Primary / 2: Secondary or SHS / 3: Polytechnic / 4: University / 5: Graduate School/ 6: Vocational training / 96: Other (Specify) |
| A07.CHW training | 0: No training / 1: CSB trained 2: NGO trained 3: Other (Specify) |
| A08.Years in current position | ________ |
| A09.About how many people live in this fokotony? | ________ |
| A10.Is there a map of all of the households in the catchment area? | - Yes - No |

**B. SUPERVISION AND ASSISTANCE**

| B1 | When was the last visit from a supervisor and from which institution?  **Check all that apply and tick corresponding columns** |  | B1A.  EMAD, other MOH | B1B.  Chef- CSB | B1.C Other CSB staff | B1D. Technical assistance from NGO | B1.E Other Technical assistance (specify) |
| --- | --- | --- | --- | --- | --- | --- | --- |
|  |  | 1. 3 months |  |  |  |  |  |
|  |  | 2. 3 – 6 months |  |  |  |  |  |
|  |  | 3. > 6 months |  |  |  |  |  |
|  |  | 4. No visit (skip to 1.3) |  |  |  |  |  |
| B2 | What things related to malaria activities did the supervisor focus on during the visit?  *Do not read responses. Select all mentioned.* | 1.Malaria commodities (ACT for Infants and children, paracetamol, ORS  2.ITNs  3.Malaria reports/data (Community RMA, patient register)  4.Lab supplies (RDT)  5.Malaria case management for children < 5 years of age  6.Social and behavior change communication (SBCC)  96.Other: ________________ | | | | | |
| B3 | What are some barriers CHWs face when trying to meet the needs of this community for malaria activities?  *Do not read responses. Select all mentioned.* | 1.Work overload  2.Many malaria cases occur among individuals > 5 years of age  3.Not having enough malaria RDTs  4.Not having enough ACTs  5.Not having enough Paracetamol  6.Lack of training  7.Difficult landscape/topography of the area  8.Long distance to community members  96.Other(s): _____________ | | | | | |
| B4 | What are the challenges in collaborating with HF?  *Do not read responses. Select all mentioned.* | 1.HF does not give enough supervision  2.HF does not give enough support with diagnosing malaria  3.HF does not give enough support with treating malaria  4.HF does not give enough support with monthly report  5.HF gives too many responsibilities  6.Delay in response from the HF when asking for assistance  7.Delay in providing commodities  8.HF is difficult to reach because of landscape or distance  96.Other (specify)____________ | | | | | |

##

## **C. MALARIA CASE MANAGEMENT**

| **Testing and Diagnosis** | | | |
| --- | --- | --- | --- |
| C1 | Are children < 5 years of age who report a fever managed in the community? | 1.Yes  2.No | |
| C2 | Are children 5 and older and adults who report a fever managed in the community? | 1.Yes  2.No | |
| **Testing and diagnosis among children < 5** | | | |
| C3 | What is done when a patient/parent reports fever?  *Do not read responses. Select all mentioned.* | 1.Check temperature  2.Ask about other symptoms  3.Perform RDT  96.Other: ________________ | |
| C4 | Do CHWs have thermometers? (if yes, ask to see it) | 1.Yes, working, unverified  2.Yes, working, verified  3.Yes, but it doesn’t work  4.No | |
| C5 | How do CHWs decide if a patient is a suspect case of malaria?  *Do not read responses. Select all mentioned.* | 1.Patient reports history of fever at home  2.Patient has measured fever during visit  3.Age of patient  4.Patient reports other symptoms  5.Patient reports fever and denies other symptoms  6.Patient reports travel to high transmission region  7.Based on time of year/Patient presents during transmission season  96.Other: _____________________ | |
| C6 | Have CHWs received malaria RDT-specific trainings? | 1a.Yes  1b.If yes, when:____________________  2.No | |
| C7 | If a patient reports a fever at home but does not have one when the temperature is taken at the health center, do they need at RDT? | 1.Yes  2.No  3.It depends on the other symptoms  99.Don’t know | |
| C8 | If a patient’s RDT is positive, what are the next steps to take?  *Do not read responses. Select all mentioned.* | 1.Ask about other symptoms  2.Give ACTs  3.Give paracaetamol  4.Give Oral Rehydration Salts  5.Ask the mother to bring the child back if he/she is not better  6.Refer to health facility if patient reports symptoms of severe malaria (convulsions, altered consciousness abnormal breathing, vomiting/diarrhea)  7.No action is needed  96.Other: ______________ | |
| C9 | What are the signs/symptoms a patient has severe disease?  *Do not read responses. Select all mentioned.* | 1.Convulsions  2.Altered consciousness  3.Abnormal breathing  4.Vomiting  5.Diarrhea  96.Other:_________________ | |
| C10 | If a patient has signs of severe disease, what actions are performed?  *Do not read responses. Select all mentioned.* | 1.Perform RDT, if positive, treat with ACT, send home  2.Perform RDT, if positive, treat with ACT and refer to the HF  3.Perform RDT, if positive, refer to the HF without ACT treatment  4.Perform RDT, if negative, treat with ACT and send home  5.Perform RDT, if negative, treat with ACT and refer to the HF  6.Perform RDT, if negative, refer to HF without ACT treatment  7.Immediately send patient to the HF without testing or treatment  8.Perform RDT then send patient to the HF regardless of result, without malaria treatment  96.Other: __________________ | |
| C11 | Please describe the system for referring severe cases to the CSB.  *Do not read responses. Select all mentioned.* | 1.All patients who need care I can’t provide are referred to the affiliated CSB  2.Very sick patients are referred to the closest hospital  3.I have funds pay for patient transport  4.I do not have funds to transport patients  5.I can call the CSB to ask for assistance transporting patients  6.Ask my CHW colleague to assist with referral decision making if she/he is available  96.Other _________________________________________ | |
| C12 | What are the challenges CHWs experience with diagnosing malaria cases?  *Do not read responses. Select all mentioned.* | 1.There are no challenges with diagnosing malaria cases  2.RDT stockouts  3.Lack of training  4.Lack of confidence in accuracy of RDT result  96.Other(s):_______________ | |
| C13 | If a patient has a positive RDT, do CHWs ask if they traveled outside of the fokontany in the previous two weeks? | 1.Yes  2.No  3a.Sometimes  3b(specify) _____________ | |
| **Comfort with Testing and Diagnosing Malaria among Older Children and Adults** | | | |
| C14 | Could CHWs treat adolescents and adults for uncomplicated malaria? | 1.Yes, if they had proper training and tools  2.No, they would not be comfortable treating older children  3.No, they do not have time for additional CHW work  99.Don’t know | |
| C15 | Could CHWs treat children aged 5–13 for uncomplicated malaria | 1.Yes, if they had proper training and tools  2.No, they would not be comfortable treating older children  3.No, they do not have time to treat older children  99.Don’t know | |
| **Malaria Treatment Activities among Children < 5** | | | |
| C16 | What are the challenges with treating malaria cases?  *Do not read responses. Select all mentioned.* | 1.There no challenges with treating malaria cases  2.ACT stock outs  3.Lack of training  4.Lack of treatment adherence (people do not take full treatment of ACTs)  5.Patient’s sharing personally prescribed antimalarial medication (with family member and others)  6.People seek treatment with traditional healers  7.Power shortage  8.Clean water shortage  96.Other(s):____________ | |
| C17 | Do CHWs follow-up on each malaria case to ensure that treatment was completed and to address potential complications and side effects? | 1.Yes  2.No  3.Sometimes (Specify) | |
| C18 | Have **you** received malaria **case management** specific trainings in the last 2 years?  Please provide details on the **last malaria case management training you** received. | 1.Yes | 1A. Number of trainings on **malaria** **case management you** received within the last 2 years? |
|  |  |  | 1B. Month(s) and Year (s)_____________ |
|  |  | 2.No (skip to section D) | |
| C19 | Do CHWs have the tools they need to implement the recommendations made during the **case management** trainings? | 1.Yes  2.No  99.Don’t know | |
| C20 | What additional malaria prevention and case management trainings would be helpful?  *Do not read responses. Select all mentioned.* | 1.No additional trainings would be useful  2.LLIN use  3.Malaria diagnosis for children under 5  4.Malaria treatment for children under 5  5.Referral of severe and complicated malaria  6.Integrated Community Case Management (iCCM)  96.Other(s): _________________ | |
| **Comfort with providing additional malaria treatment** | | | |
| C21 | If rectal medicine for severe malaria among children <5 were available, would CHWs be comfortable administering it? | 1.Yes  2.No  99.Don’t know | |
| C22 | Would CHWs be able to give more than one medicine to treat malaria (e.g. ACT plus primaquine?) | 1.Yes, if they had proper training and commodities  2.No, they would not be comfortable giving two medications  3.No, they do not have time for additional CHW work  99.Don’t know | |

## **D. SURVEILLANCE, DATA MANAGEMENT, MONITORING AND EVALUATION (SM&E)**

| **DATA MANAGEMENT AND DATA USE** | | |
| --- | --- | --- |
| D1 | Do CHWs keep a registry of all patient visits? | 1.Yes, verified  2.Yes, unverified  3.No  99.Don’t know |
| D2 | Do CHWs check the quality of the information in their patient register? | 1.Yes, they review their information every 1-2 days  2.Yes, they review their information every week  3.Yes, they review their information every month  4.Yes, they review their information occasionally  5.No  99.Don’t know |
| D3 | If no, why not?  *Do not read responses. Select all mentioned.* | 1.They have not received training  2.They don’t know how to do it  3.It did not occur to them to do it  4.It is not necessary to review the information in the register |
| D4 | Do CHWs have SOPs/guidelines for checking the quality of the information the patient register? | 1.Yes, verified  2.Yes, unverified  3.No  99.Don’t know |
| D5 | Do CHWs keep a community monthly summary report? | 1.Yes  2.No  99.Don’t know (specify)____________________________ |
| D6 | Have **CHWs** received training in recording, processing, or reporting of health information (**data management**) in the last 2 years? | 1.Yes  2.No |
| D7 | How do CHWs report data?  *Do not read responses. Select all mentioned.* | 1.They fill out community-RMAs  2.Someone from CSB comes to fill out a community RMA form  96.Other:________________ |
| D8 | Who helps CHWs with reporting | 1.No one  2.Chef CSB  3.NGO staff  96.Other |
| D9 | What types of data do CHWs report?  *Do not read responses. Select all mentioned.* | 1.Number of all consults  2.Number of children < 5 with fever  3.Number of children < 5 tested with RDT  4.Number of children < 5 with positive RDTs  5.Number of children < 5 treated with ACT  6.Number of children < 5 followed-up for completion of treatment  7.Number of RDTs currently in stock  8.Number of ACTs currently in stock  9.Number of people referred to CSB  10.Number of cases of severe malaria  11.Number of deaths due to malaria  12.Number of cases of malaria who traveled outside of the fokontany  96.Other: _______________ |
| D10 | To whom do CHWs report data?  *Do not read responses. Select all mentioned.* | 1.Chef CSB  2.CSB manager  3.District health officer  4.Non-governmental Organization or other partner  96.Other: ____________ |
| D11 | How often do CHWs report data? | 1.Weekly  2.Monthly  3.Quarterly  4.Twice a year  5.Annually  96.Other: _____________ |
| D12 | What obstacles do CHWs face sending reports on time? | 1.No funds for transportation  2.No time to complete the forms  3.No time to take the reports to the CSB  4.They don’t understand how to complete the forms  96.Other: _____________ |
| D13 | Do CHWs ever receive feedback on the data they report? | 1.Yes  2.No  3.Sometimes  99.Don’t know |
| D14 | In the past 3 months, did you receive any feedback on **malaria case management** indicators? | 1. Yes, Verbal  2. Yes, Written  3. Yes, Phone call  4. Yes, Other  5.No |
| D15 | How was the feedback beneficial?  *Do not read responses. Select all mentioned.* | 1.By showing existing gaps  2.Find solutions for gaps identified  3.Provided service improvement  4.Serve as baseline for improvement  5.Feedback was not beneficial  96.Other(s): ____________________ |
| D16 | What are some improvements, CHWs would like to see with the current malaria case reporting systems?  *Do not read responses. Select all mentioned.* | 1.No improvement is needed  2.Make the reporting form shorter  3.Collect information more often  4.Collect information less often  5.CSB should send someone to fill out the form  6.Support electronic data collection  96.Other: _______________________ |
| **DATA ANALYSIS** | | |
| D17 | What do CHWs do if they notice an increase in community members with fever?  *Do not read responses. Select all mentioned.* | 1.Consult with their CHW colleague  2.Notify the chef du village  3.Notify the supervisor by text  4.Notify the supervisor by telephone  5.Notify the supervisor by sending a community member to alert him/her  96.Other (specify)___________________________________ |
| D18 | Do CHWs use the community RMA to make decisions?  (e.g.: do CHWs look at the RMA and compare it to earlier months to see if there are increases in some conditions?) | 1.Yes  2.No (go to question D20)  96.Other (specify)___________________________________ |
| D19 | What types of decisions have you made with this data?  *Do not read responses. Select all mentioned.* | 1.Examining performance by looking at target goals and actual performance from month to month  2.Mobilizing/shifting of resources based on needs of the community  3.Asking for more malaria support by showing gaps in ability to meet target (example: by requesting for more commodities)  96.Others: ___________ |
| D20 | What are the reasons CHWs don’t use malaria data to make decisions?  *Do not read responses. Select all mentioned.* | 1.They don’t know how to do this  2.The supervisor makes these decisions  3. CHWs do not have the time to look at the data  96.Other(s): _________________ |
| D21 | What do CHWs need to improve the current malaria surveillance system(s)?  *Do not read responses. Select all mentioned.* | 1.Additional trainings  2.Additional funds Improved data recording  3.Provide reporting forms  4.Provide recording books  5.Provide data analysis tools (epidemic monitoring chart)  6.Additional help from the community  7.Strengthening health education  8.Supportive supervision  96.Other(s): _____________________________ |
| **EPIDEMIC RESPONSE** | | |
| D22 | Are CHWs able to tell if family members or neighbors have malaria at the same time? | 1.Yes  2.No  99.Don’t know |
| D23 | If multiple people who live near each other (e.g. neighbors), or many people in one family, have malaria, what would CHWs do?  *Do not read responses. Select all mentioned.* | 1.They test children < 5 for fever and malaria if they come for a visit  2.They go into the community but only test children < 5 for fever and malaria  3.They go into the community test everybody for fever and malaria  4. They go into the community and test children < 5. If people >=5 years of age have a fever, they are referred to the CSB  96.Other__________________________________ |
| D24 | What are CHWs able to do to respond to an increase in malaria cases?  *Do not read responses. Select all mentioned.* | 1.They do not have any capacity to respond to an increase in malaria cases  2.They do not typically have enough commodities to treat 2 times or more than the usual number of malaria cases among children < 5  3.They can make emergency requests for malaria commodities  4.They can receive emergency commodities within a short period  5.They can refer severe malaria cases to other HFs/ hospitals for advanced care  6.They can contact partners and request assistance to respond to malaria case increases  7.They can perform education outreach activities in the community  8.They can go into the community to test and treat/active surveillance  9.They can distribute LLINs  96.Other(s):___________________________________ |
| D25 | Have CHWs received any guidance on how to immediately respond to a malaria outbreak? | 1a.Yes,  1b. Who provided this guidance (MOH or partner)____________  2.No  99.Don’t know |
| D26 | Have you responded to an outbreak in the past two years? | 1.Yes  2.No |
| D27 | What are the challenges CHWs face when responding to outbreaks?  *Do not read responses. Select all mentioned.* | 1.There are no challenges when responding to outbreaks  2.Households are hard to reach  3.People with fever do not come to the CHW  4.People with fever seek care from traditional healers  5.Difficulty maintaining RDTs and ACTs stock at the CHW level  96.Other(s):_________________ |

## **E. MALARIA COMMODITIES**

| E1 | Which of the following are available at your facility?  *Do not read responses. Select all mentioned.* | 1.RDT  2.ITN/LLIN  3.Lancet  4.Cotton  5.Alcohol  6. Sharps container  7. Disinfectant for cleaning  8.Artesunate-Amodiaquine (AS+AQ) |
| --- | --- | --- |
| E2 | Who provides CHWs with malaria RDTs and with ACTs?  *Do not read responses. Select all mentioned.* | 1.CSB  2.District office  3.Regional Office  4.DLP  5.Partners: ______________________________  6. No one  96. Other ___________________________ |
| E3 | How often do CHWs receive RDTs and ACTs?  *Do not read responses. Select all mentioned.* | 1.As needed when they are requested  2.Weekly  3.Monthly  4.Quarterly  5.Twice a year  6.Annually  96.Other:_____________ |
| E4 | Are there two months stock of RDTs and anti-malarial drugs currently? | 1.Yes  2.No  99.Don’t know |
| E5 | Were there any RDT stock-outs in the past 3 months? | 1.Yes  2.No  99.Don’t know |
| E6 | Were there any medication stock outs in the past 3 months? | 1.Yes  2.No (go to question E8)  99.Don’t know |
| E7 | If yes, which medications were stocked-out?  *Do not read responses. Select all mentioned.* | 1.Artesunate-Amodiaquine (AS+AQ)  2.Artemether-Lumefantrine (CoArtem)  3.Quinine  4.Paracetamol  96.Other ______________ |
| E8 | Have there been stock-outs in the past 3 months of anything else such as lancets, alcohol swabs, or cotton? | 1.Yes  2.No  99.Don’t know |
| E9 | Are there currently enough malaria stocks to respond to any malaria outbreak? | 1.Yes  2.No  99.Don’t know |
| E10 | How do CHWs determine if there are enough stocks for a malaria outbreak?  *Do not read responses. Select all mentioned.* | 1.Based on number of malaria cases  2.By guessing  3.Based on malaria epidemic preparedness plan  96.Other(s):_______________________ |
| E11 | How do CHWs request/receive enough malaria commodities to be able to respond to an outbreak?  *Do not read responses. Select all mentioned.* | 1.They request extra malaria commodities before the major transmission season  2.They request an emergency supply of commodities from the CSB  96.Other(s): _______________________ |
| E12 | What actions do CHWs take when there are low stocks of malaria commodities?  *Do not read responses. Select all mentioned.* | 1.There are no actions to take when stocks are low  2.They send a request to the CSB  3.They make calls to the CSB  96.Other(s): _______________ |
| E13 | Which of the following challenges to commodity delivery do CHWs experience? | 1.There are no challenges experienced  2.Transportation challenges to the CSB from the district  3.Transportation challenges to the community from the CSB  4.Staff shortage  5.Difficult topography  6.Distance to the CSB is far  7.Storage facilities (not being available or having a space shortage)  96.Other(s): ___________________ |
| E14 | Do CHWs have a system for keeping track of commodities in stock? | 1.Yes  2.No (go to question E16) |
| E15 | If yes, how do CHWs keep track of commodities? | 1.They use the community RMA  2.They use the health visit registry  96.Other (s) (specify________) |
| E16 | If no, why not?  *Do not read responses. Select all mentioned.* | 1.It is not necessary to report stock  2.There is no form provided to record  3.CHWs have not been trained to manage commodities  96.Other(s):______________________ |
| E17 | Have **you** received **malaria commodity** specific trainings in the last 2 years? | 1.Yes  2.No |
| E18 | What additional malaria trainings should CHWs receive? | 1.Managing commodities  2.How to complete the patient registry  3.How to complete community RMA  4.How to use records to make decisions  5.Strengthening health education  6.Supportive supervision  96.Other(s): _____________________________ |

## **F. SOCIAL BEHAVIOR CHANGE COMMUNICATION AND COMMUNITY PARTICIPATION**

| **F1** | Do CHWs conduct malaria education in their communities? | 1.Yes  2.No  99.Don’t know |
| --- | --- | --- |
| **F2** | What types of malaria education efforts have CHWs conducted?  *Do not read responses. Select all mentioned.* | 1.There are no educational efforts conducted  2.House to house health education  3.Community education  4.Printed health education materials  5.Health education at community centers  96.Other(s):________________________________ |
| **F3** | What are some challenges with implementing education efforts? | 1.People are not interested  2.People don’t have time  3.CHWs don’t have adequate training  4.CHWs don’t have adequate tools  96.Other(s):________________________________ |
| **F4** | How do CHWs reach special populations like pregnant women and people who travel to areas with a lot of malaria | 1.CHWs meet with pregnant women to remind them to go to the CSB for prenatal care  2.CHWs remind travelers to consider malaria if they develop a fever  3.CHWs do not have time for programs with special populations  4.CHWs do not have training to work with special populations  96.Other(s):________________________________ |

Do you have any other comments about CHW malaria work? ___________________________________

# TOOL 6: STAKEHOLDER INTERVIEW GUIDE – at elimination districts only

**Section 1: Demographic Information (see codes below)**

| **Participant code** | **Education** | **Title** | **Years in current position** |
| --- | --- | --- | --- |
|  |  |  |  |

**Code:**

| Participant Code | Region, District, sub-district, participant category, specific participant Code (additional information on how to collect this code will be attached to this guide) |
| --- | --- |
| Education | 0: No education / 1: Primary / 2: Secondary or SHS / 3: Polytechnic / 4: University / 5: Graduate School / 6: Vocational training / 96: Other (specify):________________ |
| Title | (please write in) |
| Years in current position | 1: Less than 1 year / 2: 1–2 years / 3: more than 2 years / 96: Other (specify):_________________ |

**NATIONAL, REGIONAL, DISTRICT LEVEL**

| ***Malaria case management*** | | |
| --- | --- | --- |
| 1 | What are the main bottlenecks in successfully implementing malaria control activities? | |
| 2 | What do you suggest to overcome them? | |
| 3 | What are some strategies to maintain malaria testing for all fevers by health workers when malaria incidence is very low? | |
| 4 | What are some strategies to extend access to testing and treatment for all age groups? | |
| 5 | What practices and procedures are in place to target high-risk populations such as migrant workers and pregnant women? | |
| ***Surveillance, Monitoring, and Evaluation*** | | |
| 6 | What are some challenges in achieving high quality, routine **malaria** **surveillance** (in this area)? | |
| 7 | What do you think is needed to improve the current malaria surveillance system(s)?  *Do not read responses. Select all mentioned.* | 1.Additional trainings  2.Budget support  3.Improve data recording  4.Provide reporting forms  5.Provide recording books  6.Provide data analysis tools (epidemic monitoring chart)  7.Additional staff  8.Strengthening health education in the community  9.Supportive supervision  96.Other(s): _____________________________ |
| 8 | What is the process for evaluating the quality of routine data? | |
| 9 | What are some suggestions for improving the quality of routine data? | |
| ***Supply chain*** | | |
| 10 | Do health facilities use stock management tools? | 1.Yes  2.No  99.Don’t know |
| 11 | What are the protocols in place for treatment of malaria if there is a medication stockout? | |
| 12 | Are there procedures for Health Facilities to proactively request more commodities? | 1.Yes  2.No  99.Don’t know |
| 13 | If so, how do Health Facilities request more commodities? | |
| 14 | Are there procedures for CHWs to pro-actively request more commodities? | 1.Yes  2.No  99.Don’t know |
| 15 | If so, how do CHWs request more commodities? | |
| ***Epidemic/Outbreak response*** | | |
| 16 | How are epidemic thresholds determined? | |
| 17 | At what level of the health system is an epidemic threshold set?  *Do not read responses. Select all mentioned.*  *(note to interviewer: this question does not ask the level at which the decision is made)* | 1.At the regional level  2.At the district level  3.At the sub-district level  4.At the fokontany level |
| 18 | How often are the thresholds adjusted? | 1.Yearly  2.Every 6 mo  3.Every 3 mo  4.Every month  96.Other:________ |
| 19 | Are there guidelines or SOPs for malaria outbreak response? | 1.Yes  2.No  99.Don’t know |
| 20 | Who develops these plans?  *Do not read responses. Select all mentioned.* | 1.National malaria control program  2.Regional staff  3.District staff  4.Health facility staff  96.Other:__________ |
| 21 | Who is responsible for coordinating these efforts and implementing the plan? | 1.National malaria control program  2.Regional staff  3.District staff  4.Health facility staff  96.Other:__________ |
| 22 | What kind of strategy is in place when an epidemic threshold is reached?  *Do not read responses. Select all mentioned.* | 1.SBCC outreach  2.Community-level test and treat (active case detection)  3.LLIN distribution  4.IRS campaign  5.Rapid response teams or other assistance from outside the area  6.Increased frequency of data collection and reporting  96.Other:_____________ |
| 23 | In what ways are Health Facilities prepared to implement these actions/plans?  *Do not read responses. Select all mentioned.* | 1.Training  2.Supportive supervision  3.Simulation exercises  4.Reading materials/bulletins  5. Buffer or emergency commodities/stocks  96.Other:_______________ |
| 24 | If CHWs participate in epidemic response, in what ways are they prepared to implement these actions/plans?  *Do not read responses. Select all mentioned.* | 1.Trainings  2.Supportive supervision  3.Simulation exercises  4.Reading materials/bulletins  5. Additional commodities  96.Other:_______________ |
| ***Elimination*** | | |
| 25 | Based on 2016 data, there are 5 elimination and 3 pre elimination districts identified in Madagascar. How do you define a pre elimination/elimination phase? | |
| 26 | What specific plans, guidelines and targets are available or planned for malaria elimination at national level? | |
| 27 | Please describe how areas are chosen for elimination activities. | |
| 28 | What elimination training and support is currently being offered or is planned for targeted districts? | |
| 29 | Is staff turnover a barrier to achieving malaria control and elimination?  *Do not read responses. Select all mentioned.* | 1.Health facility staff turnover is a barrier  2.CHV turnover is a barrier  3.Staff turnover is not a barrier  96. Other_________ |
| 30 | How many more and what type of staff do you anticipate needing to support malaria elimination or response activities? | |
| 31 | What are some retention and motivation strategies being used among health facility workers and CHWs? | |
| 32 | What are some suggestions for including the private sector in elimination efforts? | |
| 33 | What are some ways to adapt the routine data collection and reporting system to support elimination activities? | |
| ***Recommendations*** | | |
| 34 | Other general comments (*overall needs for future interventions to improve the management and surveillance of malaria cases? - External and environmental factors, any recommendations)* | |
