# Supplementary material for: Malaria case management and elimination readiness in health facilities of five districts of Madagascar in 2018
Source: Malar J. 2020 Oct 1;19:351. doi: 10.1186/s12936-020-03417-z (PMC7528237; doi:10.1186/s12936-020-03417-z)
Supplement: Supplementary file 4 — Additional file 4: Table S2. Malaria elimination readiness score and individual domain scores by facility system (out of 100). [file 12936_2020_3417_MOESM4_ESM.docx]

Additional Table 2. Malaria elimination readiness score and individual domain scores by facility system (out of 100)

| Health Facility | Resource Availability | Case Management | Data Management and Use | Training, Supervision, and Assistance | Total |
| --- | --- | --- | --- | --- | --- |
| **Antsiranana II** | | | | | |
| Health Facility 1 | 67 | 32 | 70 | 58 | 57 |
| Health Facility 2 | 72 | 50 | 63 | 58 | 61 |
| Health Facility 3 | 64 | 48 | 57 | 57 | 57 |
| Health Facility 4 | 64 | 33 | 74 | 52 | 56 |
| Health Facility 5 | 67 | 56 | 63 | 52 | 59 |
| Health Facility 6 | 67 | 36 | 71 | 42 | 54 |
| Health Facility 7 | 72 | 45 | 77 | 34 | 57 |
| **Antsiranana I** | | | | | |
| Health Facility 8 | 64 | 53 | 66 | 62 | 61 |
| Health Facility 9 | 78 | 60 | 57 | 45 | 60 |
| Health Facility 10 | 44 | 52 | 63 | 33 | 48 |
| Health Facility 11 | 69 | 56 | 40 | 21 | 47 |
| **Mahajanga I** | | | | | |
| Health Facility 12 | 44 | 47 | 77 | 33 | 50 |
| Health Facility 13 | 50 | 44 | 59 | 51 | 51 |
| Health Facility 14 | 69 | 053 | 61 | 18 | 50 |
| Health Facility 15 | 50 | 46 | 53 | 57 | 51 |
| Health Facility 16 | 50 | 55 | 58 | 57 | 55 |
| Health Facility 17 | 39 | 62 | 64 | 67 | 58 |
| **Antsirabe II** | | | | | |
| Health Facility 18 | 61 | 54 | 72 | 51 | 59 |
| Health Facility 19 | 61 | 039 | 63 | 27 | 48 |
| Health Facility 20 | 46 | 50 | 61 | 23 | .45 |
| **Antananarivo Atsimondrano** | | | | | |
| Health Facility 21 | 78 | 31 | 90 | 47 | 61 |
| Health Facility 22 | 67 | 57 | 65 | 41 | 58 |
| Health Facility 23 | 56 | 48 | 82 | 37 | 56 |
| Health Facility 24 | 56 | 46 | 71 | 44 | 54 |
| Health Facility 25 | 56 | 50 | 77 | 18 | 50 |
| Health Facility 26 | 56 | 36 | 59 | 52 | 51 |
| Health Facility 27 | 56 | 41 | 82 | 20 | 49 |
| Health Facility 28 | 75 | 30 | 52 | 41 | 49 |
| Health Facility 29 | 44 | 47 | 52 | 43 | 47 |
| Health Facility 30 | 56 | 27 | 65 | 36 | 46 |
| Health Facility 31 | 44 | 35 | 72 | 31 | 46 |
| Health Facility 32 | 61 | 53 | 45 | 19 | 45 |
| Health Facility 33 | 50 | 46 | 52 | 27 | 44 |
| Health Facility 34 | 50 | 36 | 39 | 19 | 36 |
| Health Facility 35 | 46 | 41 | 21 | 11 | 30 |
